# The triglyceride glucose-body mass index: a non-invasive index that identifies non-alcoholic fatty liver disease in the general Japanese population

**DOI:** 10.1186/s12967-022-03611-4

**Published:** 2022-09-05

**Authors:** Haofei Hu, Yong Han, Changchun Cao, Yongcheng He

**Affiliations:** 1 Department of Nephrology, The First Affiliated Hospital of Shenzhen University, Shenzhen, 518000 Guangdong China; 2 Department of Nephrology, Shenzhen Second People’s Hospital, Shenzhen, 518000 Guangdong China; 3 Department of Emergency, Shenzhen Second People’s Hospital, Shenzhen, 518000 Guangdong China; 4 Department of Emergency, The First Affiliated Hospital of Shenzhen University, Shenzhen, 518000 Guangdong China; 5 Shenzhen University Health Science Center, Shenzhen, 518071 Guangdong China; 6 Department of Rehabilitation, Shenzhen Dapeng New District Nan’ao People’s Hospital, No. 6, Renmin Road, Dapeng New District, Shenzhen, 518000 Guangdong China; 7 Department of Nephrology, Shenzhen Hengsheng Hospital, No. 20 Yintian Road, Baoan District, Shenzhen, 518000 Guangdong China

**Keywords:** Non-alcoholic fatty liver disease, Triglyceride glucose-body mass index, Negative predictive value, Receiver operating characteristic, Positive predictive value

## Abstract

**Background:**

By identifying individuals at high risk for non-alcoholic fatty liver disease (NAFLD), interventional programs could be targeted more effectively. Some studies have demonstrated that triglyceride glucose-body mass index (TyG-BMI) showed an independent positive association with NAFLD. However, research on its diagnostic value in patients with suspected NAFLD is limited. In this study, we aimed to evaluate whether TyG-BMI was accurate in detecting NAFLD in the general Japanese population.

**Methods:**

A cross-sectional study of 14,280 individuals who underwent a comprehensive health examination was conducted. Standard protocols were followed to collect anthropometric measurements, lab data, and ultrasonography features. All participants were randomly stratified into the development group (n = 7118) and validation group (n = 7162). The TyG-BMI was calculated. Following this, the diagnostic value of the TyG-BMI was evaluated based on the area under the receiver-operating characteristic curve (AUROC). Two cutoff points were selected and used to rule out or rule in the NALFD, and the specificity, sensitivity, negative predictive value, and positive predictive value were explored, respectively. In order to verify the stability of the results, external verification was performed.

**Results:**

There were 1272 and 1243 NAFLD participants in the development and validation groups, respectively**.** The area under the ROC curve (AUC) of TyG-BMI was 0.888 (95% CI 0.876–0.896) and 0.884 (95% CI 0.875–0.894) for the training and validation group, respectively. Using the low TyG-BMI (182.2) cutoff, NAFLD could be excluded with high accuracy (negative predictive value: 96.9% in estimation and 96.9% in validation). The presence of NAFLD could effectively be determined by applying the high cutoff of TyG-BMI (224.0), as the positive predictive value of the estimation and validation groups is 70.7% and 70.1%, respectively. As a result of applying this model, 9996 (70%) of the 14,280 participants would not have undergone ultrasonography, with an accurate prediction of 9308 (93.1%). AUC was 0.874 for external validation using 183,730 Chinese non-obese participants. TyG-BMI was demonstrated to be an excellent diagnostic tool by both internal and external validation.

**Conclusions:**

In conclusion, the present study developed and validated a simple, non-invasive, and cost-effective tool to accurately separate participants with and without NAFLD in the Japanese population, rendering ultrasonography for identifying NAFLD unnecessary in a substantial proportion of people.

**Supplementary Information:**

The online version contains supplementary material available at 10.1186/s12967-022-03611-4.

## Introduction

A non-alcoholic fatty liver disease (NAFLD) is marked by hepatic steatosis without evidence of excessive alcohol use or other obvious factors that damage the liver [1]. In the 21st century, NAFLD remains an essential public health issue [2]. Globally, an estimated 20% of the general population is suffering from NAFLD, with a range from 6 to 35% based on multiple measurements [3]. There is a continuum of NAFLD, from simple steatosis to non-alcoholic steatohepatitis (NASH), with varying degrees of fibrosis that eventually progress to cirrhosis [4, 5]. NASH may cause cirrhosis and hepatocellular carcinoma, while simple steatosis presents as a benign condition with slow progression over many years [3, 6, 7]. The extrahepatic form of NAFLD is characterized by its ability to aggravate the cardiovascular disease, kidney disease, and diabetes, resulting in adverse health effects [8–10]. Even though the prevalence of NAFLD is increasing and its adverse effects are seen throughout multiple systems in the body, there are no effective treatments except for lifestyle changes along with regular physical activity [11]. It is therefore extremely important to identify patients whose risk of NAFLD is high at an early stage.

As far as diagnosis of NAFLD is concerned, liver biopsy has always been the gold standard [12]. However, due to its invasiveness and high cost, it could not become a widely accepted diagnosis. It is also unreasonable to perform routine liver biopsies as a screening or risk assessment test for the general population. In addition, a liver biopsy analysis with poor inter-observer variability and modest intra-observer variability has suboptimal reliability for measuring a treatment effect in clinical trials [13]. Clinical practitioners use liver ultrasound as a valuable tool in their practice for detecting fatty liver in the early stages. Ultrasonography (US), nevertheless, depends on the operator's experience and technological sophistication [14]. Besides, Steatosis less than 20% [15] or steatosis in morbidly obese individuals could not be detected by ultrasound [16]. In addition, the accuracy of US for hepatic steatosis assessment is affected by the presence of severe fibrosis [17]. Moreover, there is a drawback in that dietary and pharmacological interventions are unable to be qualitatively evaluated [18]. With the development of ultrasonic transient elastography, the controlled attenuation parameters of the liver and the liver stiffness value can be used to assess the degree of hepatic steatosis and fibrosis quantitatively, but they are affected by the operator’s skill level [19]. In addition, based on proton magnetic resonance spectroscopy (MRS) and magnetic resonance imaging (MRI), which can accurately determine the amount of liver fat content and the degree of fibrosis, and play the role of similar liver biopsy, but their cost is high, and difficult to obtain, so it has not been widely used clinically [20]. In recent years, serum non-invasive diagnostic markers or models have attracted widespread clinical attention due to their advantages of non-invasiveness, low cost, simple operation, strong reproducibility, and low requirements for operators, especially for early screening and evaluation of NAFLD [20]. Therefore, effective noninvasive methods should be used in clinical practice to identify NAFLD, track disease processes, and monitor treatment effects [21].

Overweight, obesity, and insulin resistance strongly correlate with NAFLD due to excessive fat accumulation, especially triglycerides in hepatocytes [22]. NAFLD is characterized by oxidative stress and inflammation. An increase in reactive oxygen species (ROS) can lead to lipid peroxidation by damaging both membrane structure and function. In addition to oxidizing key proteins for cell metabolism and function, it may also cause the oxidation of nucleic acids [23]. Since the liver has a limited ability for triglyceride accumulation, lipid deposition under overfeeding conditions, as in the case of NAFLD, determines the accumulation of high levels of fatty acids, generally saturated ones, which are associated with cell dysfunction [24]. Indeed, the excess of fatty acids induces high rates of β-oxidation, increasing the production of ROS in the mitochondrial respiratory chain, which causes cellular damage and oxidative stress [25]. Oxidative damage markers rise in response to this circumstance, Kupffer cells become active, pro-inflammatory pathways activate, and circulating immune cells are drawn into the body [26, 27].

An insulin resistance (IR) is characterized by decreased peripheral tissue insulin sensitivity, which is at the core of the pathogenesis of NAFLD by impairing glucose uptake and oxidation [11, 28]. Triglyceride-glucose (TyG) is an index combining fasting blood glucose (FPG) with fasting triglyceride (TG) that could better reflect insulin resistance. It has been widely accepted and used in clinical applications due to its convenience and simple calculation [29–31]. Using a combination of body mass index (BMI) and TyG index, Er et al. found that the information imparted by a multitude of critical clinical indicators could be simultaneously reflected, such as blood lipids, blood glucose, and BMI, and could better reflect IR than the TyG index alone [32]. Given the importance of IR in NAFLD pathogenesis [11, 28], TyG-BMI has been linked to an increased incidence of NAFLD, according to certain studies [33–36]. As a result, we hypothesized that the TyG-BMI might be an effective marker in identifying NAFLD in the general population. However, as a non-invasive and simple model, applying TyG-BMI to identify and evaluate NAFLD still needs further research.

The objective of this study was to determine the diagnostic accuracy of the TyG-BMI in detecting NAFLD in the general Japanese population.

## Methods and materials

### Study population and design

In this study, the TyG-BMI was tested for its ability to detect NAFLD in the general Japanese population in a cross-sectional design. As a secondary analysis, we used data derived from a published article shared by Takuro Okamura et al. [37]. We obtained the data from the ‘DATADRYAD’ database (https://datadryad.org/stash/). This website permitted users to freely download the raw data. Dryad is a nonprofit membership organization that is committed to making data available for research and educational reuse now and into the future. According to Dryad Terms of Service, we cited the Dryad data package in the present study. (Dryad data package: Okamura, Takuro et al. Data from: ectopic fat obesity presents the greatest risk for incident type 2 diabetes: a population-based longitudinal study, Dryad, Dataset, https://doi.org/10.5061/dryad.8q0p192) [38]. From 2004 to 2015, the original study enrolled 20,944 participants ≥ 18 years of age who had at least two routine physical examinations at Murakami Memorial Hospital.

The database file contains the following variables: waist circumference (WC), gamma-glutamyltransferase (GGT), gender, total cholesterol (TC), age, diastolic blood pressure (DBP), smoking status, BMI, aspartate aminotransferase (AST), ethanol consumption, TG, alanine aminotransferase (ALT), high-density lipoprotein cholesterol (HDL-c), FPG, systolic blood pressure (SBP), hemoglobin A1C (HbA1c), comorbidity with fatty liver and the habit of exercise.

**Fig. 1:**
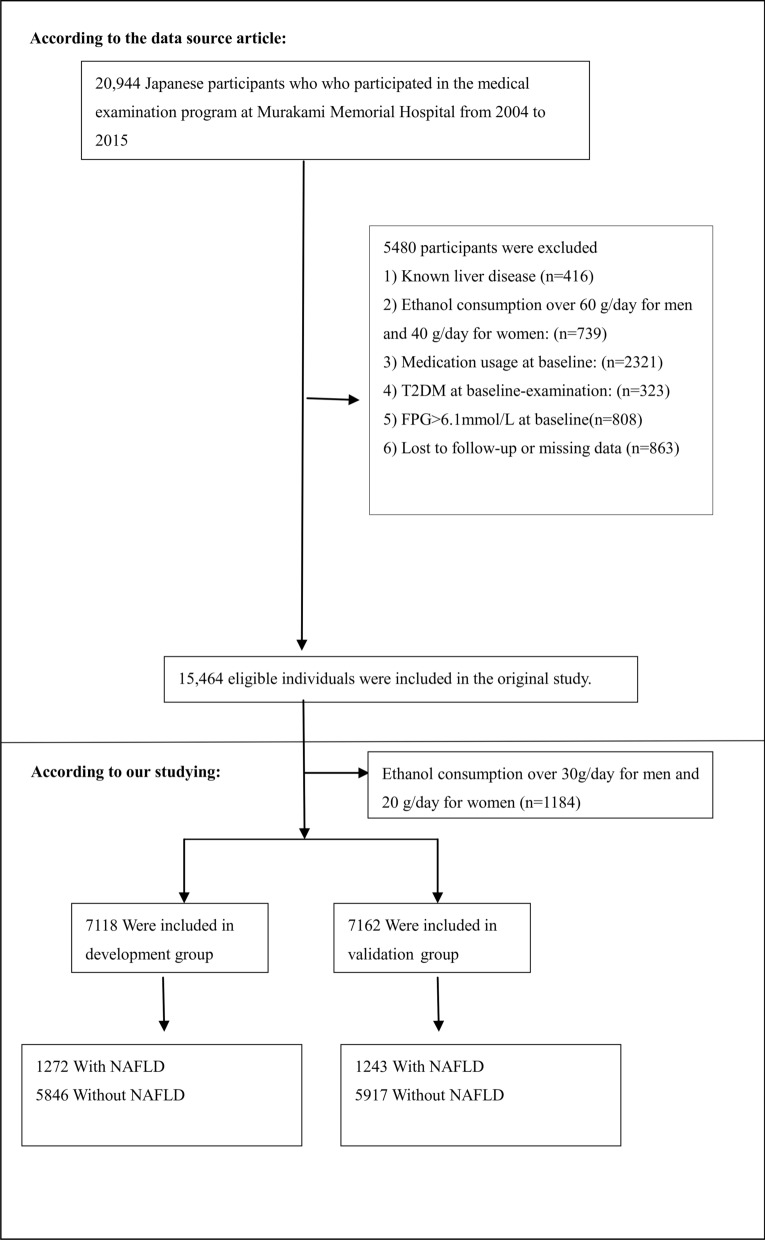
Flowchart of study participants. The inclusion of participants. The eligibility of 15,464 participants was assessed in the original study. We excluded individuals with ethanol consumption over 30 g/day for men and 20 g/day for women (n = 1184). In the present study, 14,280 subjects were included in the final analysis

Exclusion criteria of the original study included: (1) participants diagnosed with type 2 diabetes (n = 323) or with fasting plasma glucose (FPG) over 6.1 mmol/L (n = 808); (2) participants with known liver disease, such as hepatitis B or C virus (n = 416); (3) anyone who took medication (n = 2321); (4) participants with heavy drinking habits (more than 40 g per day for women and more than 60 g per day for men) (n = 739); (5) participants with a missed value of covariates, including abdominal ultrasonography, exercise, alcohol intake or laboratory variables (n = 863) [37]. A total of 15,464 participants were included in the raw study for the final analysis. 1184 participants in the present study were further excluded for excessive alcohol consumption (in males > 210 g/week and in females > 140 g/week) [39]. Figure 1 showed the process of selecting participants. Finally, 14,280 subjects (7440 males and 6840 females) were included in this secondary analysis.

Murakami Memorial Hospital’s Ethics Committee approved the research ethics, and all subjects provided informed consent in the original study [37].

### Health check-ups and laboratory measurement

A standard and unified questionnaire was used by trained medical staff to gather basic health information about the subjects, including height, habit of exercise, weight, blood pressure (SBP and DBP), WC, age, smoking and drinking status. Biochemical analysis of blood samples was conducted after at least 8 h of fasting. Analysis indicators included HbA1c, ALT, FPG, TG, HDL-c, GGT, TC, and AST [37].

#### Definitions and calculations

BMI = weight divided by height^2^. TyG = Ln [(FPG (mg/dL)/2) × TG (mg/dL)] [30]. TyG-BMI = BMI × TyG [32]. Ethanol consumption was evaluated by the mean ethanol intake of participants per week during the prior month. The smoking status was categorized into current smokers, ex-smokers, or non-smokers. According to World Health Organization 2020 guidelines on physical activity, regular exercise was defined as follows: adults should undertake 150–300 min of moderate-intensity, or 75–150 min of vigorous-intensity physical activity, or some equivalent combination of moderate-intensity and vigorous-intensity aerobic physical activity, per week [40].

### Diagnosis of NAFLD by abdominal ultrasonography

An abdominal ultrasound was used to assess NAFLD, and gastroenterologists, without knowledge of the participants' personal information, reviewed the ultrasound images. The final diagnosis was made based on the evaluation of four ultrasound findings: liver brightness, liver, and kidney echo contrast, vessel blurring, and depth attenuation [41]. This was a new scoring system of ultrasonographic findings in apparently healthy Japanese adults. The AUC to diagnose NAFLD was 0.980. The sensitivity was 91.7% (95% CI 87.0–95.1), and the specificity was 100% (95% CI 95.4–100.0).

### Statistical analysis

A random stratification process was used to divide participants into training and validation groups. For continuous variables, the mean (standard deviation) was given for normal distribution, the median (range) for non-normal distribution, and the number (%) for categorical variables. The authors used the student’s *t*-test (normal distribution), the *χ*^2^ (categorical variables), or the Mann–Whitney’s *U*-test test (non-normal distribution) to test for differences between development and validation groups. Stratified by the presence of NAFLD, the authors also showed the characteristics of the validation and training groups, respectively.

Using the area under the receiver operating characteristic (ROC) curve (AUC) and its 95% confidence intervals, the overall diagnostic accuracy of the TyG-BMI was determined in the development and validation groups, respectively. Using 500 bootstrap resamplings, the authors computed the AUC with a 95% CI with TyG-BMI to evaluate its discriminatory properties and validate its diagnostic accuracy [42].

Through the ROC curve, 2 cutoff points were selected according to the deciles of TyG-BMI and considering the specificity (SP), sensitivity (SE), negative predictive value (NPV), and positive predictive value (PPV) of the two cutoff points. The two cutoff points were used to rule out or rule in the NALFD, respectively. The authors calculated specificity, sensitivity, NPV, PPV, positive likelihood ratios (PLR), and negative likelihood ratios (NLR) to determine the diagnostic accuracy of the two cutoff points. The authors also explored the cutoff points' diagnostic values for different NAFLD prevalence or different gender and age subgroups.

Moreover, the authors used a database of 183,730 general Chinese populations for external validation. The data were also taken from the DATADRYAD database (https://datadryad.org/stash), shared by Sun et al. [43]. Data from: association of low-density lipoprotein cholesterol within the normal range and NAFLD in the non-obese Chinese population: a cross-sectional and longitudinal study, Dryad, Dataset, https://doi.org/10.5061/dryad.1n6c4. All participants were non-obese people with a normal range of Low-density lipoprotein cholesterol (LDL-c), as described in the original article [43]. Decision curve analysis was performed to explore the clinical use of TyG-BMI for the diagnosis of NAFLD: the proportion of people who showed a true positive result was first subtracted from those who showed a false positive result, then weighed against the relative risks of false-positive and false-negative results, and finally, obtained the net benefit of making a decision [44].

All results were reported according to the STARD statement [45]. All analyses were carried out using statistical packages from the R (http://www.r-project.org, The R Foundation) and EmpowerStats packages (http://www.empowerstats.com, X and Y Solutions, Inc, Boston, MA). P values less than 0.05 were considered statistically significant (two-sided).

## Results

In the present study, 14,280 participants (52.1% men and 47.9% women) were eligible. Figure 1 depicted the subjects’ selection and grouping process. The mean age of all participants was 43.53 ± 8.89 years. A total of 2515 (17.6%) participants were diagnosed with NAFLD. The mean BMI was 22.07 ± 3.14 kg/m^2^. The mean FPG and TG were 92.74 ± 7.42 and 79.03 ± 56.07 mg/dL, respectively. The mean TyG and TyG-BMI were 8.01 ± 0.64 and 177.74 ± 34.53, respectively.

### Baseline characteristics of participants

Table 1 illustrated the eligible participants' basic demographic and clinical information. The authors randomly divided all participants into the development group (n = 7118) and the validation group (n = 7162). 1272 and 1243 participants were diagnosed with NAFLD in the development and validation groups, respectively. In all baseline characteristics, the development group did not differ statistically from the validation group (all P > 0.05).

**Table 1:** Baseline characteristics of the development and validation groups

Characteristic	Development group	Validation group	P-value
N	7118	7162	
Age (years)	43.533 ± 8.918	43.533 ± 8.864	0.964
Alcohol consumption (g/w)			0.893
= 0	2364 (33.212%)	2371 (33.105%	
> 0	4754 (66.788%)	4791 (66.895%)	
BMI (kg/m^2^)	22.046 ± 3.104	22.090 ± 3.168	0.398
WC (cm)	76.199 ± 9.026	76.193 ± 9.174	0.968
ALT (U/L)	17.000 (13.000–23.000)	16.000 (12.000–23.000)	0.161
AST (U/L)	17.000 (14.000–21.000)	17.000 (14.000–21.000)	0.727
GGT (U/L)	15.000 (11.000–21.000)	15.000 (11.000–21.000)	0.830
HDL-c (mmol/L)	1.462 ± 0.405	1.455 ± 0.399	0.285
TC (mmol/L)	5.125 ± 0.867	5.123 ± 0.869	0.901
TG (mmol/L)	0.723 (0.485–1.095)	0.723 (0.485–1.106)	0.291
TyG-BMI	177.386 ± 34.108	178.093 ± 34.940	0.222
HbA1c (%)	5.176 ± 0.321	5.180 ± 0.321	0.470
FPG (mmol/L)	5.145 ± 0.414	5.152 ± 0.410	0.308
SBP (mmHg)	113.878 ± 14.774	114.043 ± 14.892	0.506
SBP (mmHg)	71.031 ± 10.376	71.251 ± 10.407	0.204
SEX, n (%)			0.604
Female	3394 (47.682%)	3446 (48.115%)	
Male	3724 (52.318%)	3716 (51.885%)	
Regular exerciser, n (%)	1239 (17.407%)	1237 (17.272%)	0.831
Smoking status, n (%)			0.934
Never-smoker	4369 (61.380%)	4382 (61.184%)	
Ever-smoker	1284 (18.039%)	1288 (17.984%)	
Current-smoker	1465 (20.582%)	1492 (20.832%)	

Values are n (%) or mean ± SD or medians (quartiles)

*HDL-c* high-density lipoprotein cholesterol, *ALT* alanine aminotransferase, *FPG* fasting plasma glucose, *TC* total cholesterol, *DBP* diastolic blood pressure, *HbA1c* hemoglobina1c, *AST* aspartate aminotransferase, *BMI* body mass index, *TG* triglyceride, *SBP* systolic blood pressure, *GGT* gamma glutamyltransferase, *WC* Waist circumference, *TyG-BMI*: triglyceride glucose-body mass index

By NAFLD status, Table 2 showed the characteristics of the 2 groups. The participants with NAFLD had higher BMI, WC, alcohol consumption, SBP, age, FPG, DBP, TG, HbA1c, ALT, TC, AST, BUN, GGT, and higher rates of males and ever or current smokers in the development and validation groups. In contrast, participants in the NAFLD group had lower levels of HDL-c.

**Table 2:** Baseline characteristics for the training and validation groups by NAFLD status

Characteristic	Development group		Validation group	
	Non-NAFLD	NAFLD	Non-NAFLD	NAFLD
N	5846	1272	5919	1243
Age (years)	43.314 ± 9.050	44.537 ± 8.215	43.219 ± 8.923	45.027 ± 8.427
Alcohol consumption (g/w)				
= 0	1992 (34.075%	372 (29.245%)	1997 (33.739%)	374 (30.088%)
> 0	3854 (65.925%)	900 (70.755%	3922 (66.261%)	869 (69.912%)
BMI (kg/m^2^)	21.305 ± 2.581	25.448 ± 3.037	21.363 ± 2.633	25.551 ± 3.215
WC (cm)	74.090 ± 7.851	85.890 ± 7.636	74.117 ± 7.990	86.078 ± 7.920
ALT (U/L)	15.000 (12.000–20.000)	27.000 (20.000–38.000)	15.000 (12.000–20.000)	27.000 (20.000–39.000)
AST (U/L)	14.000 (11.000–18.000)	20.000 (16.000–25.000)	17.000 (14.000–20.000)	21.000 (17.000–26.000)
GGT (U/L)	14.000 (11.000–18.000)	22.000 (16.000–32.250)	14.000 (11.000–18.000)	23.000 (16.000–33.000)
HDL-c (mmol/L)	1.524 ± 0.402	1.181 ± 0.280	1.512 ± 0.394	1.186 ± 0.300
TC (mmol/L)	5.065 ± 0.859	5.397 ± 0.851	5.046 ± 0.847	5.488 ± 0.882
TyG-BMI	168.514 ± 27.726	218.162 ± 30.871	169.266 ± 28.289	220.124 ± 32.960
TG (mmol/L)	0.655 (0.452–0.937)	1.231 (0.858–1.727)	0.655 (0.452–0.960)	1.264 (0.881–1.829)
HbA1c (%)	5.148 ± 0.312	5.303 ± 0.329	5.155 ± 0.312	5.299 ± 0.339
FPG (mmol/L)	5.092 ± 0.405	5.386 ± 0.368	5.099 ± 0.400	5.404 ± 0.359
SBP (mmHg)	111.875 ± 14.020	123.082 ± 14.665	111.991 ± 14.031	123.811 ± 15.000
DBP (mmHg)	69.607 ± 9.894	77.572 ± 10.025	69.814 ± 9.830	78.097 ± 10.356
SEX, n (%)				
Female	3153 (53.934%)	241 (18.947%)	3209 (54.215%)	237 (19.067%)
Male	2693 (46.066%)	1031 (81.053%)	2710 (45.785%)	1006 (80.933%)
Regular exerciser, n (%)	1040 (17.790%)	199 (15.645%)	1058 (17.875%)	179 (14.401%)
Smoking status, n (%)				
Never-smoker	3777 (64.608%)	592 (46.541%)	3788 (63.997%)	594 (47.788%)
Ever-somker	966 (16.524%)	318 (25.000%)	964 (16.287%)	324 (26.066%)
Current-smoker	1103 (18.868%)	362 (28.459%)	1167 (19.716%)	325 (26.146%)

Values are n (%) or mean ± SD or medians (quartiles)

*HDL-c* high-density lipoprotein cholesterol, *BMI* body mass index, *ALT* alanine aminotransferase, *FPG* fasting plasma glucose, *TC* total cholesterol, *DBP* diastolic blood pressure, *HbA1c* hemoglobina1c, *AST* aspartate aminotransferase, *TG* triglyceride, *SBP* systolic blood pressure, *WC* waist circumference, *GGT* gamma-glutamyltransferase, *TyG-BMI* triglyceride glucose-body mass index

**Fig. 2:**
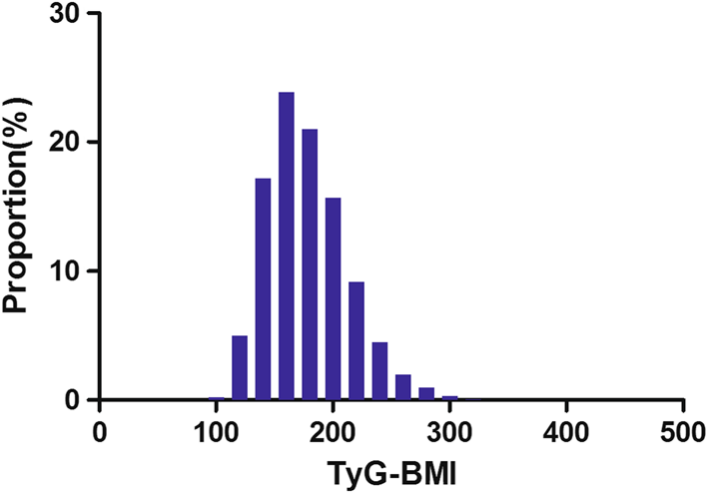
Distribution of TyG-BMI. TyG-BMI presented a normal distribution ranging from 97.49 to 421.35 in the total population, with a mean level of 177.74

**Fig. 3:**
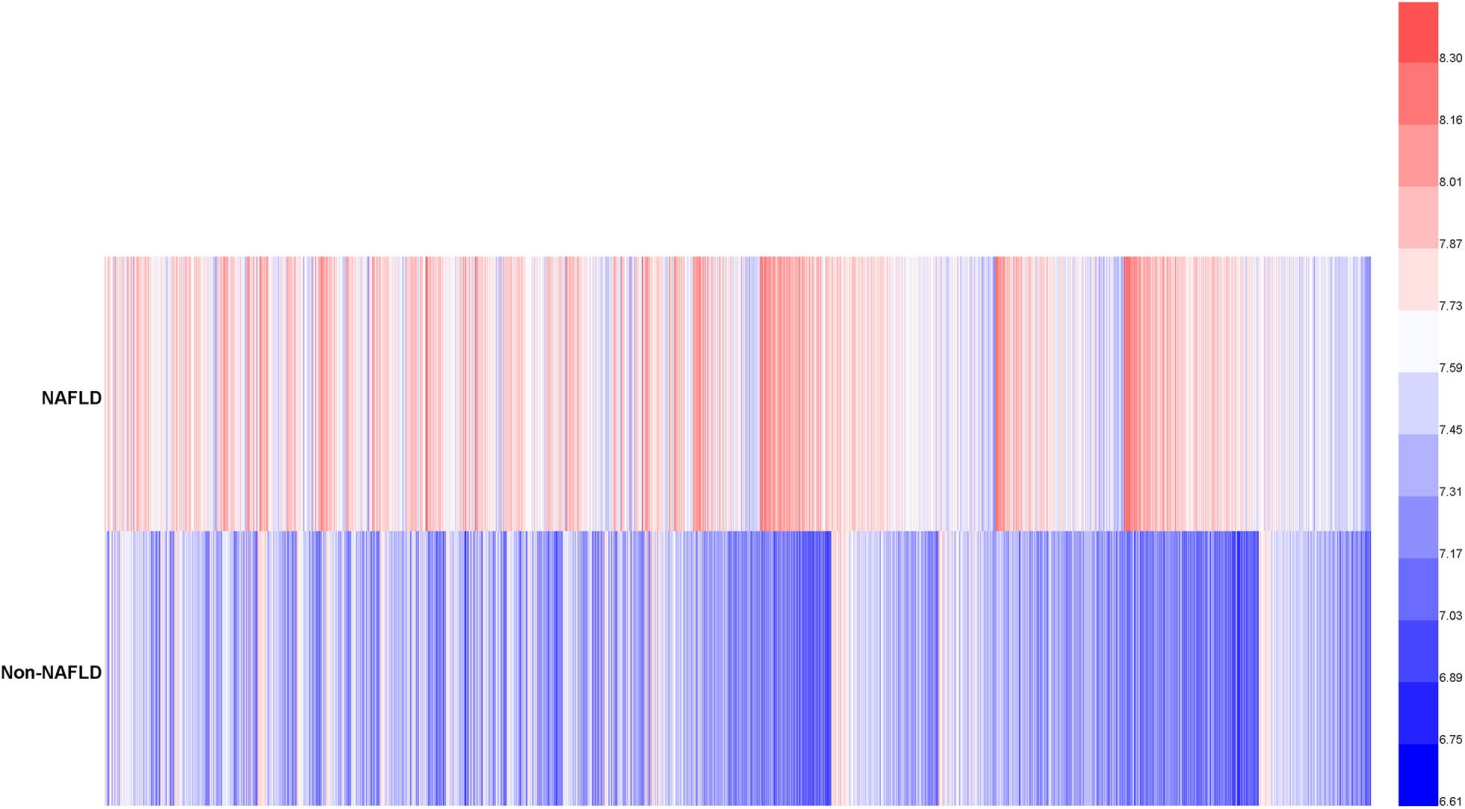
Data visualization of TyG-BMI of all participants from the NAFLD and non-NALFD groups. The TyG-BMI distribution level in the NAFLD group was higher than the TyG-BMI level in the non-NAFLD group

**Fig. 4:**
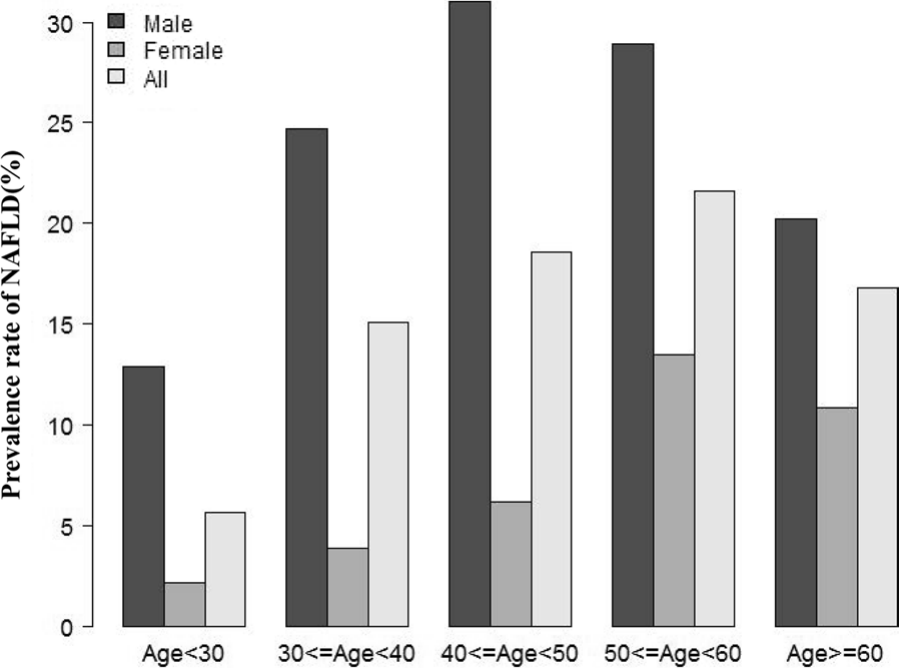
NAFLD prevalence of age stratification by 10 intervals. Men were found to have a higher prevalence of NAFLD in age-stratified by 10 intervals than women, regardless of age group. Meanwhile, the study also found that the prevalence of NAFLD increased stepwise in both male (except for those older than 50) and female (except for those older than 60) participants with increasing age

TyG-BMI levels were distributed in a normal distribution, as shown in Fig. 2 and Additional file 1: Fig. S1. They ranged from 97.49 to 421.35 in the total population. The TyG-BMI values of all the participants from the NALFD and non-NAFLD groups were shown in Fig. 3. As a result, the distribution level of TyG-BMI was higher in the NALFD group than in the non-NALFD group. Men were found to have a higher prevalence of NAFLD in age-stratified by 10 intervals than women, regardless of age group (Fig. 4). Meanwhile, the study also found that the prevalence of NAFLD increased stepwise in both male (except for those older than 50) and female (except for those older than 60) participants with increasing age (Fig. 4). All participants were divided into four groups according to quartiles of TyGBMI, and we found that participants with a high TyGBMI had higher prevalence rates of NAFLD compared to the group with the lowest TyGBMI (P < 0.0001 for trend) (Additional file 2: Fig. S2).

### Development phase

**Fig. 5:**
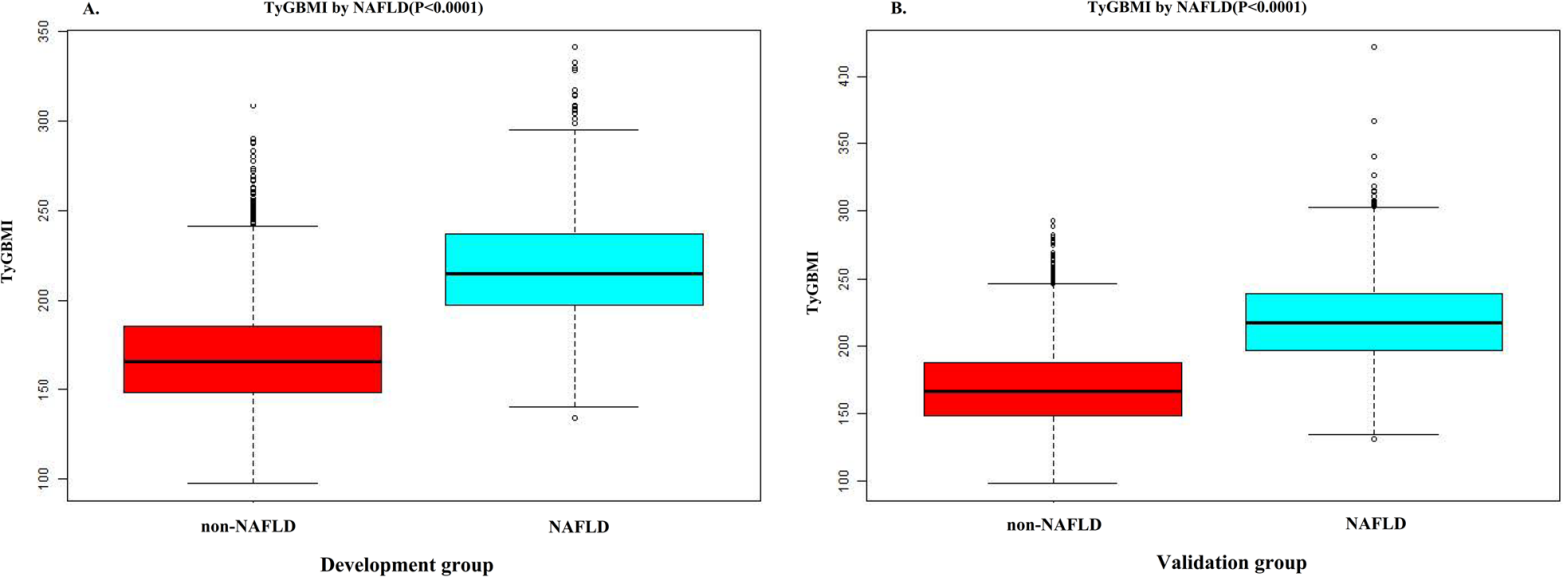
TyG-BMI for participants with and without NAFLD in the development and validation groups. **A** indicated that the median TyG-BMI was elevated among participants with NAFLD (214.5) compared to those without NAFLD (166.2). **B** indicated that the median TyG-BMI was also elevated among participants with NAFLD (217.1) compared with 166.6 participants without NAFLD. Boxes have bottom and top edges representing first and third quartiles, respectively. The band within the box is the median value, while the whiskers represent values that are 1.5 times the interquartile range.

**Fig. 6:**
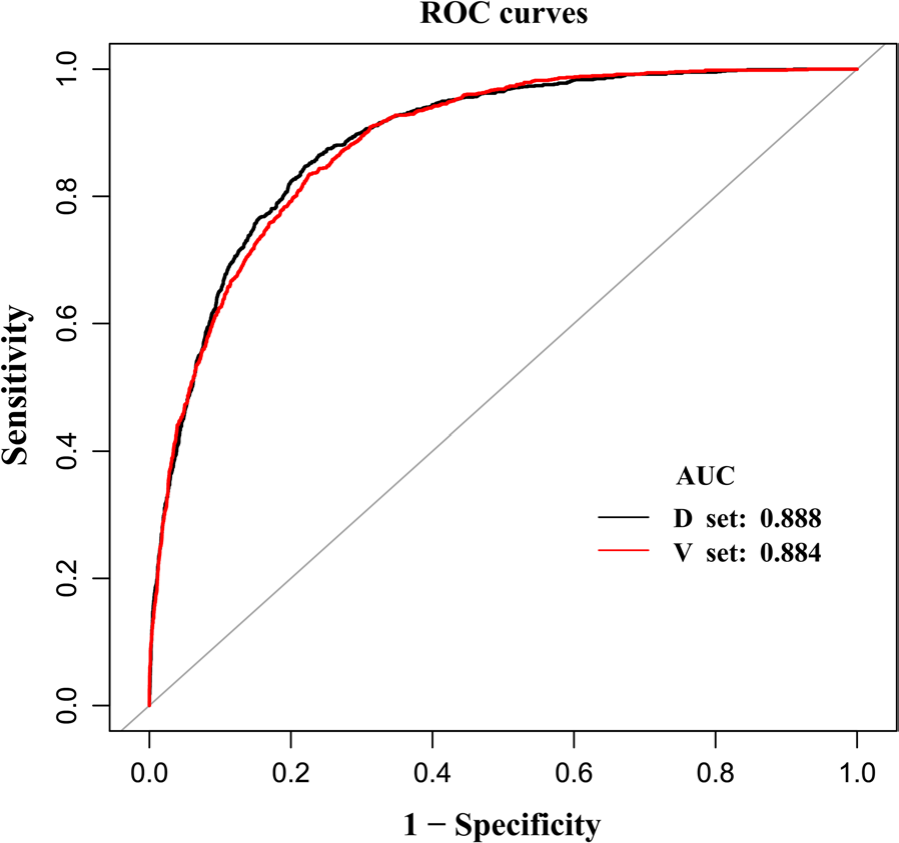
The ROC curve of the modeling group and validation group. The diagnostic accuracy of TyG-BMI in separating participants with and without NAFLD was analyzed by using the ROC method. The AUC remained high in the development group [0.888 (95% CI 0.879, 0.897)] and in the validation set [0.884 (95% CI 0.875, 0.894)]

The median TyG-BMI was elevated among participants with NAFLD (214.5). For participants without NAFLD, the median level was 166.2 (Fig. 5A). The authors applied the ROC method to analyze the diagnostic accuracy of the TyG-BMI for detecting NAFLD in the development group. TyG-BMI had an AUC of 0.888 (95% CI 0.879, 0.897) (Fig. 6, Additional file 7: Table S1). Using 500 bootstrap resamplings, TyG-BMI had an average AUC of 0.886 (95% CI 0.876, 0.896). The AUROC remained high and almost unchanged in the development set (Additional file 3: Fig. S3).

Table 3 described the diagnostic accuracy of TyG-BMI in predicting NAFLD at decile intervals. In the development group, when the cut-off point of the TyG-BMI was set at 182.2 to discriminate NAFLD, it would meet the relatively high Youden’s index (0.605) and the diagnostic accuracy of sensitivity (89.4%)/specificity (71.1%), PPV (40.2%)/NPV (96.9%), and LR + (3.09)/LR−(0.15). Meanwhile, when the cut-off point of the TyG-BMI was set at 224.0, the diagnostic accuracy of sensitivity (38.1%)/specificity (96.6%), PPV (70.7%)/NPV (87.8%), and LR + (11.09)/LR−(0.64). So a TyG-BMI < 182.2 could be used to rule out (SE = 89.4%, NPV = 96.9% LR− = 0.15) and a TyG-BMI ≥ 224.0 to rule in NAFLD (SP = 96.6%, PPV = 70.7%, LR +  = 11.1) (Table 3).

**Table 3:** Diagnostic accuracy of the TyG-BMI

	Cut-off	No	SP (%)	SE (%)	PPV (%)	NPV (%)	PLR	NLR	Youden’s index
Development	≥ 137.4	6405	12.2	99.9	19.8	99.9	1.14	0.006	0.121
	≥ 147.8	5705	24.2	99.5	22.2	99.6	1.31	0.020	0.237
	≥ 156.7	4998	35.9	98.5	25.1	99.1	1.54	0.042	0.344
	≥ 165.0	4264	48.2	97.1	29.0	98.7	1.87	0.060	0.453
	≥ 173.3	3541	60.0	94.3	33.9	98.0	2.36	0.094	0.543
	≥ 182.2	2829	71.1	89.4	40.2	96.9	3.09	0.149	0.605
	≥ 192.8	2113	81.2	79.6	48.0	94.8	4.25	0.251	0.608
	≥ 205.4	1396	90.2	64.9	59.1	92.2	6.64	0.389	0.551
	≥ 224.0	686	96.6	38.1	70.7	87.8	11.09	0.641	0.347
Validation	≥ 137.4	6447	12.1	99.8	19.3	99.7	1.14	0.013	0.119
	≥ 147.8	5719	24.4	99.6	21.7	99.7	1.32	0.017	0.240
	≥ 156.7	4998	36.3	99.0	24.6	99.4	1.55	0.029	0.353
	≥ 165.0	4304	47.8	97.6	28.2	98.9	1.87	0.050	0.454
	≥ 173.3	3599	59.1	94.3	32.6	98.0	2.30	0.097	0.534
	≥ 182.2	2883	70.1	89.2	38.5	96.9	2.98	0.154	0.593
	≥ 192.8	2171	80.0	79.2	45.5	94.8	3.97	0.260	0.592
	≥ 205.4	1460	89.0	65.2	55.4	92.4	5.92	0.391	0.542
	≥ 224.0	742	96.3	41.8	70.1	88.7	11.18	0.605	0.381

*PPV* positive predictive value, SP specificity, *NPV* negative predictive value, *SE* sensitivity, *PLR* positive likelihood ratio, *NLR* negative likelihood ratio, *TyG-BMI* triglyceride glucose-body mass index

Using a low cutoff point (below 182.2), 4156 (71.1%) of the 5846 individuals without NAFLD were correctly identified, whereas 135 (3.3%) of 4289 individuals with a low cutoff point were incorrectly identified (Table 4). Thus, this low cutoff point could exclude the absence of NAFLD with high accuracy (NPV of 97%).

**Table 4:** The diagnostic value of TyG-BMI obtained from the development and validation group

	Low cutoff point (< 182.2)	Indeterminate (182.2–224.0)	High cutoff point (> 224.0)	Total
Development				
Total	4289	2143	686	7118
Non-NAFLD	4156	1489	201	5846
NAFLD	135	652	485	1272
Sensitivity	89.4%		38.1%	
Specificity	71.1%		96.6%	
PPV	40.4%		70.7%	
NPV	96.9%		87.8%	
PLR	3.09		11.09	
NPR	0.15		0.64	
Interpretation	Absence of NALFD (97% certainty)		Presence of NAFLD (71% certainty)	
Validation				
Total	4279	2141	742	7162
Non-NAFLD	4148	1550	221	5919
NAFLD	134	590	519	1243
Sensitivity	89.2%		41.8%	
Specificity	70.1%		96.3%	
PPV	38.5%		70.1%	
NPV	96.9%		88.7%	
PLR	2.98		11.18	
NLR	0.15		0.61	
Interpretation	Absence of NALFD (97% certainty)		Presence of NAFLD (70% certainty)	

*PPV* positive predictive value, *NPV* negative predictive value, *PLR* positive likelihood ratio, *NLR* negative likelihood ratio, *TyG-BMI* triglyceride glucose-body mass index

By applying the high cutoff point (above 224.0), 485 (38.1%) of 1272 participants with NALFD were correctly identified, whereas 201 (29%) of the 686 with the high cutoff point were incorrectly staged (Table 4). With this high cutoff point, it was possible to diagnose NALFD with high accuracy (71% PPV for detection).

Overall, in the development group, TyG-BMI predicted the absence or presence of NAFLD in (4289 + 686)/7118 = 70% of participants with a correct diagnosis in 4641/4975 or 93% [or 65% (4641/7118) of the total]. The incorrect diagnosis rate in the development group was only (135 + 201)/4975 = 6.75%. As a result, 4975 (70%) participants would have avoided abdominal ultrasonography if the model had been applied to the development group. Only 2143 (30%) of the 7118 participants with “indeterminate” status (TyG-BMI in the range of 182.2–224.0) would need to undergo ultrasound imaging (Table 4).

Significant differences in NAFLD prevalence were found in age and gender stratification. The diagnostic performance of the TyG-BMI in different gender and age strata for NAFLD was also evaluated using ROC analysis. TyG-BMI showed a larger AUC for distinguishing NAFLD in females, young, and middle-aged people (Table 5).

**Table 5:** Performance of the tests for diagnosis/exclusion of NAFLD by different subgroups

Development group	AUROC (95% CI)	Cutoff	SE (%)	SP (%)	PPV (%)	NPV (%)	PLR	NLR
Sex								
Male	0.84 (0.83–0.86)	182.2	91.1	55.4	43.9	94.2	2.04	0.16
		224.0	39.3	94.6	73.5	80.3	7.25	0.64
Female	0.92 (0.92–0.93)	182.2	82.2	84.6	28.9	98.4	5.32	0.21
		224.0	32.8	98.3	59.3	95.3	18.79	0.68
Age (years)								
< 30	0.97 (0.94–0.99)	182.2	92.3	88.1	33.3	99.4	7.77	0.087
		224.0	53.8	99.0	77.8	97.1	54.38	0.49
30–40	0.91 (0.90–0.93)	182.2	90.9	75.9	42.2	97.7	3.77	0.12
		224.0	44.1	97.0	74.2	90.0	14.83	0.58
40–50	0.88 (0.87–0.90)	182.2	89.8	70.1	42.1	96.8	3.10	0.14
		224.0	38.8	96.2	70.1	87.1	10.34	0.64
50–60	0.85 (0.83–0.87)	182.2	87.2	63.4	38.5	95.0	2.38	0.20
		224.0	30.3	95.8	65.5	83.9	7.23	0.73
> 60	0.82 (0.76–0.89)	182.2	86.0	57.9	27.4	95.7	2.04	0.24
		224.0	28.0	86.6	66.7	88.0	10.07	0.76

*PPV* positive predictive value, *SP* specificity, *NPV* negative predictive value, *SE* sensitivity, *PLR* positive likelihood ratio, *NLR* negative likelihood ratio, *AUROC* area under the receiver-operating characteristic curve

### Validation Phase

In the validation group, the median TyG-BMI was also elevated among participants with NAFLD (217.1) compared with 166.6 among participants without NAFLD (Fig. 5B). The diagnostic accuracy of TyG-BMI in separating participants with and without NAFLD was analyzed by using the ROC method. The AUC remained high in the validation set [0.884 (95% CI 0.875, 0.894)] (Fig. 6, Additional file 7: Table S1), and also 500 bootstrap resamplings [0.886(95% CI 0.877, 0.897)] (Additional file 4: Fig. S4).

In the development group, when the cut-off point of the TyG-BMI was set at 182.2 to discriminate NAFLD, it would meet the relatively high Youden’s index (0.605) and the diagnostic accuracy of SE (89.2%)/SP (70.1%), PPV (38.5%)/NPV (96.9%), and LR + (2.98)/LR−(0.15). Meanwhile, when the cut-off point of the TyG-BMI was set at 224.0, the diagnostic accuracy of sensitivity (41.8%)/specificity (96.3%), PPV (70.1%)/NPV (88.7%), and LR + (11.18)/LR−(0.61). So a TyG-BMI < 182.2 could be used to rule out (SE = 89.2%, NPV = 96.9% LR− = 0.15) and a TyG-BMI ≥ 224.0 to rule in NAFLD (SP = 96.3%, PPV = 70.1%, LR +  = 11.2) (Table 3).

By applying the low cutoff point (below 182.2), 4148 (70.1%) of the 5919 participants without NAFLD were correctly identified, whereas 134 (3.1%) of 4279 with a low cutoff point were incorrectly staged (Table 4). Thus, this low cutoff point could also exclude the absence of NAFLD with high accuracy (NPV of 96.9%).

By applying the high cutoff point (greater than 224.0), 519 (41.8%) of the 1243 participants with NAFLD were correctly identified, whereas only 221 (29.9%) of the 742 participants with a high cutoff point were incorrectly staged (Table 4). It was possible to detect the presence of NAFLD with high accuracy using this high cutoff point (PPV of 70.1%).

Overall, in the validation group, the model identified the absence or presence of NAFLD in (4279 + 742 = 5021)/7162 = 70% of participants with a correct diagnosis in 4667/5021 = 93% [or 65.2% (4667/7162) of the total]. The incorrect diagnosis rate in the validation group was only (134 + 221)/5021 = 7.1%. Therefore, abdominal ultrasonography could have been avoided in 5021 (70%) of the participants if the model had been used in the validation group. Only 2141 (30%) of the 7162 participants identified as “indeterminate” (TyG-BMI in the range of 182.2–224) would receive ultrasonography.

The authors also found that TyG-BMI had a larger AUC to distinguish NAFLD in female and young and middle-aged people in the validation group (Additional file 7: Table S2).

### Predictive values of the TyG-BMI for different prevalence of NAFLD

The worldwide prevalence of NAFLD ranges from 6 to 35% [3]. As a result, the authors calculated the positive and negative predictive values of the two cutoff points using a range of prevalences of NAFLD ranging between 5 and 50%. The NPV of the low cutoff point to rule out NAFLD decreased as the prevalence of NAFLD increased, but it remained high (≥ 87.8%, Table 6) when the prevalence of NAFLD was less than 30%. The PPV of the high cutoff point to diagnose NALFD increased as the prevalence of NAFLD increased. It also remained high, particularly for the prevalence of 20% or more (≥ 73.7%, Table 6). Thus, these two cutoff points may be helpful in diagnosing NAFLD in participants with different prevalences of NAFLD.

**Table 6:** Diagnostic values of the cut-off points for different prevalences of NALFD

Prevalence of NAFLD (%)	Lower Cutoff Value (< 182.2)		Higher Cutoff Value (> 224.0)	
	PPV (95% CI)	NPV (95% CI)	PPV (95% CI)	NPV (95% CI)
5	26.1 (21.1–31.8)	98.3 (97.6–98.9)	37.1 (27.9–47.2)	96.7 (95.8–97.4)
10	42.7 (35.2–50.6)	96.5 (95.0–97.6)	55.5 (43.0–67.2)	93.4 (91.5–94.8)
15	54.2 (45.3–62.9)	94.6 (92.2–96.3)	66.4 (52.6–78.0)	89.8 (87.1–92.1)
20	62.6 (53.0–71.4)	92.5 (89.3–94.9)	73.7 (59.3–84.5)	86.2 (82.6–89.2)
25	69.1 (59.1–77.6)	90.3 (86.2–93.3)	77.9 (63.4–88.0)	82.4 (77.9–86.1)
30	74.2 (64.1–82.3)	87.8 (82.8–91.6)	82.8 (68.2–91.8)	78.5 (73.2–83.0)
35	78.3 (68.2–86.0)	85.2 (79.2–89.7)	86.1 (71.7–94.2)	74.3 (68.3–79.6)
40	81.7 (71.7–88.9)	82.3 (75.3–87.6)	88.2 (73.9–95.5)	70.1 (63.2–76.1)
45	84.6 (74.7–91.2)	79.1 (71.2–85.3)	90.2 (76.1–96.7)	65.6 (58.1–72.4)
50	87.0 (77.3–93.1)	75.6 (66.7–82.8)	91.8 (78.0–97.6)	60.1 (52.9–68.5)

*PPV* positive predictive value, *NPV* negative predictive value

### External validation

The external validation was performed on a database of 183,730 Chinese non-obese participants with a normal range of LDL-c. The mean age, BMI, TG, and FPG of the participants were 40.98 ± 14.06 years old, 21.43 ± 2.13 kg/m^2^, 118.43 ± 90.39 mg/dL, and 92.76 ± 15.33 mg/dL, respectively (Additional file 7: Table S3). The AUC of the external validation was 0.874 (Additional file 5: Fig. S5). The NPV, sensitivity, and specificity rate of the low cutoff point to rule out NAFLD were 98.5%, 94.1%, and 60.0%, respectively. While the PPV, specificity, and sensitivity rate of the high cutoff point to diagnose NALFD were 64.0%, 97.9%, and 23.6%, respectively (Table 7). The external validation revealed that TyG-BMI's ability to diagnose NAFLD could be promoted to some extent.

**Table 7:** Diagnostic value of the TyG-BMI from the external verification data

	Low cutoff point (< 182.2)	Indeterminate (182.2–224.0)	High cutoff point (> 224.0)	Total
Total	96453	77897	9380	183730
Non-NAFLD	94958	59911	3374	158243
NAFLD	1495	17986	6006	25487
Sensitivity	94.1%		23.6%	
Specificity	60.0%		97.9%	
PPV	27.5%		64.0%	
NPV	98.5%		88.8%	
PLR	2.35		11.05	
NLR	0.098		0.78	
Interpretation	Absence of NALFD (98.5% certainty)		Presence of NAFLD (64% certainty)	

*PPV* positive predictive value, *NPV* negative predictive value, *PLR* positive likelihood ratio, *NLR* negative likelihood ratio, *TyG-BMI* triglyceride glucose-body mass index

### Clinical use of the model

**Fig. 7:**
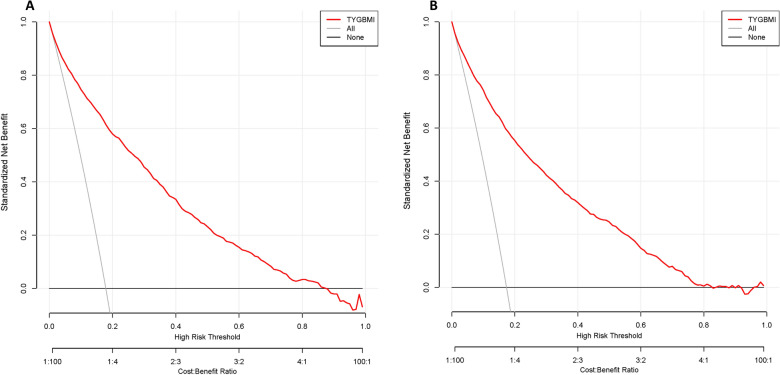
The decision curve analysis of TyG-BMI for NAFLD in the training group (**A**) and validation group (**B**). TyG-BMI had good clinical application value for diagnosing or excluding NAFLD in the training and validation groups. When none of the participants are considered to develop NAFLD, the black line represents the net benefit. When all participants are considered to develop NAFLD, the light gray line represents the net benefit. A model's diagnostic utility is defined as the distance between the "no treatment line" (black line) and the "all treatment line" (light gray line) in its curve. It is better to use TyG-BMI in clinical settings when the model curve is farther from the black line and light gray line

The decision curve analysis of the TyG-BMI was demonstrated in Fig. 7 in the training and validation groups. As it could see from the graph, the black line represented the net benefit when no participants had been diagnosed with NALFD. In contrast, the light gray line represented the net benefit when everyone had been diagnosed with NALFD. A model’s diagnostic utility was defined as the distance between the “no treatment line” (black line) and the “all treatment line” (light gray line) in its curve. In terms of clinical application, the further the model curve was from the black and light gray lines, the better. Specifically, in the training cohort, the net benefit was equal to performing 50 additional NAFLD screenings (such as abdominal ultrasonography) per 100 Japanese adults if the threshold probability was 30% in the model when without a significant change in the prevalence of NAFLD (Fig. 7A). Similar results could be obtained in the internal and external validation participants (Fig. 7B, Additional file 6: Fig. S6).

## Discussion

This cross-sectional study aimed to develop and validate a non-invasive index that uses routinely measured and readily accessible clinical and laboratory variables to discriminate between the presence or absence of NAFLD. This index, called the “TyG-BMI”, accurately distinguished the populations with or without NAFLD. The absence or presence of NAFLD was diagnosed in 9996 (70%) of the 14,280 patients using values below or above the lower or upper cutoff points. Of these 9996 individuals, 9308 (93.1%) were diagnosed correctly. Only 4284 participants (30%) of the 14,280 participants with TyG-BMI in the range of 182.2–224 were considered “indeterminate”. According to this, 70 percent (9996 out of 14,280) of participants in the whole population were able to avoid ultrasonography by applying the TyG-BMI. Both internal and external validations demonstrated that TyG-BMI was highly accurate in diagnosing patients. In addition, the authors summarized the positive and negative predictive values of the two cutoff points using a wide range of prevalence of NAFLD, ranging from 5 to 50%. TyG-BMI’s clinical application was demonstrated by the decision curve analysis.

A number of non-invasive and simple models have been developed to detect and evaluate NAFLD [18, 46]. Due to their calculation based on anthropometric and biochemical parameters, these could be easily obtained in clinical practice. Hepatic steatosis has been identified and managed with these models because they are cost-effective, practical, and reliable [46]. Several studies have shown that the fatty liver index (FLI), which is derived from a population of fewer than 8000 individuals in an Italian municipality [47], is acceptable for detecting NAFLD [48]. Based on a survey of nearly 10,000 Korean patients, the hepatic steatosis index (HSI) has also been shown to be an accurate and simple method for predicting NAFLD [49]. A few other indicators may be used to determine central lipid accumulation, including lipid accumulation product (LAP) and visceral adiposity index (VAI) [50, 51]. The underlying cause of NAFLD is a complex combination of environmental factors, heredity, and dietary habits [52]. Several dietary habits contribute to the development of NAFLD, such as excessive calorie consumption, fructose consumption, and physical inactivity [53]. Moreover, Western and Asian countries differ significantly in genetic backgrounds, dietary habits, and lifestyles [54]. It is possible, however, that these indices may not be appropriate for Asian populations since they were most originally designed for western populations. Furthermore, most centers do not have external validation of these models, making it challenging to apply the proposed scoring system daily.

In a Japanese population, Wang et al. developed a novel model called the TyG-BMI index that could help predict NAFLD [34]. After adjusting for confounding variables, according to the study, NAFLD was positively associated with TyG-BMI (OR: 3.90 per SD increase; 95% CI 3.54 to 4.29). Analysis of ROC showed that the TyG-BMI was more effective at predicting NAFLD risk than other traditional indicators [TyG-BMI (AUC): 0.886; TyG (AUC): 0.808; TG (AUC): 0.797; BMI (AUC): 0.858; FPG (AUC): 0.711], especially among young and middle-aged individuals and individuals who aren't obese. With the AUROC of 0.886 (95% CI 0.876, 0.896) in general populations and 0.88–0.97 in young and middle-aged people, and 0.84 in non-obese people (Additional file 7: Table S4), the results of the present study were consistent with Wang et al. [34]. However, hepatic steatosis commonly occurs in obese individuals. We consider the reasons for this phenomenon as follows. After further analysis of the baseline information of subjects for BMI stratification, we found that there were more non-obese women than men in this study. Sex differences in non-obese NAFLD have also been noted in some previous studies [55, 56]. There is a general tendency for females to have more subcutaneous and visceral fat [57, 58], and the BMI alone does not provide a complete picture of this information [56]. According to recent studies, people with non-obese NAFLD are more likely to develop metabolic diseases [59].

Wang et al. [59] carried out a receiver operating characteristic analysis that showed that the TyG-BMI could better predict the risk of NAFLD than other traditional indicators and obtained the optimal threshold for TyG-BMI. However, the performance of TyG-BMI has not been validated in an external population in the study of Wang et al. [34]. In addition, they did not explore two cut-off values of TyG-BMI to identify or exclude NAFLD and the corresponding positive and negative predictive values. It is essential to point out that although the optimal threshold had the largest Youden index, it is not associated with the greatest positive or negative predictive value. Therefore, a diagnostic and exclusion model of a disease requires 2 cut-off values, and the optimal threshold is not the best choice. To address this question, the present study developed and validated a simple, non-invasive, and cost-effective tool, TyG-BMI, to accurately separate participants with and without NAFLD in the Japanese population. A total of 2 cut-off values of TyG-BMI were found in this study, one for excluding NALFD and the other for diagnosing NALFD. The cut-off was 182.2 for the sensitivity of 0.894 and 224.0 for the specificity of 0.966 in the derivation cohort, leading to a negative predictive value of 0.969, a positive predictive value of 0.707, and an area under the ROC curve of 0.888 (95% CI 0.876–0.896). The results demonstrated that, as a result of applying this model, 9996 (70%) of the 14,280 participants would not have undergone ultrasonography, with an accurate prediction of 9308 (93.1%). Thereby facilitating the more accurate identification and selection of candidates for clinical intervention and reducing the number of unnecessary ultrasonography. Therefore, this model has good clinical application prospects.

In 2019, Mohammad et al. [36] developed triglyceride glucose index and related parameters (triglyceride glucose-waist circumference and triglyceride glucose-body mass index) to identify NAFLD in individuals with overweight/obesity in Iran. They found that TyG-WC showed the largest AUC for detection of NAFLD [0.693, 95% confidence interval (CI) 0.617–0.769], followed by TyG-index [0.676, 95% CI 0.598–0.754] and TyG-BMI (0.675, 95% CI 0.598–0.752). Another study [33] focuses on the association between TyG-BMI and NAFLD in the non-obese Chinese population with normal blood lipid levels. In their study, TyG-BMI had a good prediction value (0.85 area under ROC; 95% CI 0.84–0.86) for NAFLD incidence. The AUROCs of the two studies were a bit smaller than the present study. Besides, the AUROC of TyG-BMI was less than TyG for detecting NAFLD in the study of Mohammad E. et al.[36]. Several factors might explain the difference: (1) the study populations differed. The present study was performed on the general Japanese, while the above two studies focused on Iranian with overweight/obesity or the non-obese Chinese population with normal blood lipid levels. (2) The diagnosis method of NAFLD was different. There was a difference between ultrasonography and transient elastography. (3) The prevalence of NAFLD varied significantly by gender, age, dietary habits, and ethnicity [54].

Japanese dietary pattern is also different from Chinese dietary pattern. In the Japanese diet, the total energy is lower, and essential fatty acids (e.g., N-3 fatty acids) are higher because of more seafood consumption compared to the Chinese diet. NAFLD is a multifactorial disease related to a complex living environment, heredity, and dietary habits [52]. In patients with NAFLD, dietary n-3 polyunsaturated fatty acids (PUFAs) can reduce hepatic inflammation, fibrosis, and steatosis, lower plasma TG levels, and improve hepatic fatty acid metabolism [60]. Different dietary habits affected the prevalence of NAFLD in Chinese and Japanese populations, and the prevalence could affect the effectiveness of TyG-BMI in diagnosing NAFLD. However, our results validated in the Chinese population suggest that the AUC was 0.874. The results indicate that TyG-BMI has an excellent ability to identify NAFLD in both Chinese and Japanese people.

Applying the TyG-BMI index, the results of the present study suggested that ultrasonography would only be needed to identify NAFLD in 30 percent of participants, i.e., those considered “indeterminate” (TyG-BMI in the range of 182.2–224). Most importantly, since most persons seen in clinical practice were not suffering from NAFLD [82.4% (11,765/14280) of the study cohort], the lower cutoff point was exceptionally accurate in ruling it out. In both estimation and validation, the NPV was 97% and 97%, respectively, and ranged from 75.6 to 98.3% for a prevalence of NAFLD of 5–50%. Among 14,280 patients, 8568 (60%) had a negative diagnosis of NAFLD through TyG-BMI (TyG-BMI below 182.2), and thus, using the TyG-BMI would have prevented the need for ultrasound. Of these 8568 participants diagnosed as not having NAFLD by TyG-BMI, 8304 (97%) were confirmed by ultrasound to have non-NAFLD indeed.

It should be pointed out that, clinically, US is the preferred imaging test for individuals with suspected NAFLD [61], with a typical appearance of a hyperechogenic liver. In a recent meta-analysis, ultrasound showed 85% sensitivity and 94% specificity in diagnosing moderate-to-severe steatosis compared to histology [14]. In contrast, US could not detect steatosis of less than 20% [15] or steatosis in individuals with morbid obesity [16]. Moreover, ultrasound cannot determine how severe NAFLD steatosis is [17]. Using computed-assisted US hepatic/renal ratios and US hepatic attenuation rates, it is possible to detect NAFLD early [17, 62]. Compared to the conventional US, both measurements are excellent in detecting hepatic steatosis, with a sensitivity of 95% and specificity of 100%. However, the NPV is still low (72% for US H/R ratio and 67% for US hepatic attenuation rate) [17, 63]. In addition, by standardizing it with a tissue-mimicking phantom, this quantitative US model can improve its reliability and reproducibility, while these findings are needed to verify in further studies [63]. Above all, it is still recommended by current guidelines that US be used to diagnose moderate and severe steatosis [64]. Vibration-controlled transient elastography (TE) is one of the available non-invasive assessment tools for NAFLD. By generating vibrations of low frequency and mild amplitude, elastic shear waves propagate through liver tissues and are used for measuring stiffness [65]. With newer models, liver fibrosis can now be measured by liver stiffness measurement (LSM), and liver steatosis can be measured by controlled attenuation parameter (CAP) [66]. There are several benefits of TE, including its low cost, fast procedure time, immediate result availability, good reproducibility, and ability to be performed in an outpatient setting [67]. Several cross-sectional studies have investigated how it helps diagnose NAFLD and assess its severity [63, 65, 68]. In conclusion, the quantitative US model and transient elastography will become a good non-invasive method for diagnosing NAFLD in the future, as an essential improvement of traditional ultrasonography.

Lipotoxicity in hepatocytes and immune-mediated inflammation play a crucial role in the development and progression of NAFLD. Hepatocellular injury caused by the lipotoxicity of accumulated lipids and free fatty acids (FFAs) is characterized by oxidative stress, endoplasmic reticulum stress, mitochondrial dysfunction, apoptosis, and subsequent expression of pro-inflammatory cytokines and inflammatory factors [69]. Apoptotic and immune pathways are activated as a consequence of cellular injury, which represents a distinctive feature of NASH pathophysiology. The lipotoxic lipids can activate both the intrinsic- and extrinsic-mediated (death receptor) apoptotic pathway in hepatocytes through the transcriptional up-regulation of proapoptotic and downregulation of antiapoptotic proteins [70]. It is believed that apoptosis or other forms of hepatocyte cell death play a crucial role in promoting immune responses associated with the progression of NAFLD to a more severe stage, e.g., fibrosis and cirrhosis development [71].

Since oxidative stress is a significant feature of NAFLD, antioxidant therapy is of great value for NAFLD [23]. The Mediterranean diet, Silymarin and berberine could play an antioxidant role, thereby protecting liver cells [72–74]. In humans, only a small amount of oral antioxidants are absorbed because they are easily destroyed by acids and enzymes. Consequently, the development of effective methods for efficiently delivering antioxidants is urgently needed. Nano-antioxidants, created as a sponge-like polymer, act as a protective vehicle to prevent antioxidants from being degraded in the human gut and promote improved absorption in the digestive tract. A nano-capsule binds itself to the intestinal wall and releases antioxidants right into the intestinal cells, where they are absorbed directly into the bloodstream. Numerous antioxidant units are connected in a branched pattern to form nano-antioxidants. It could provide numerous possible sites to couple with an active species and have enhanced free radical scavenging potency [75].

The current study has some strengths, as follows: (1) the present study included a sizable sample size and diverse individuals, making it simple to publicize outside of the study. (2) TyG-BMI is determined using objective clinical and easily accessible lab variables routinely measured during health checkups, without requiring any other tests. (3) The authors explored two cutoff points used to identify or exclude NAFLD and used a wide range of prevalence of NAFLD varying from 5 to 50% to study the changes in positive and negative predictive values. (4) Using our decision curve analysis, TyG-BMI’s clinical effectiveness was demonstrated, and individuals with low-risk NAFLD would not require additional screening (such as ultrasonography). (5) The authors validated the results both internally and externally to make sure they were reliable.

Despite TyG-BMI’s good performance, the study still has some potential limitations. First, due to its imperfect sensitivity, ultrasonography is not a gold standard in diagnosing NALFD. The liver biopsy in asymptomatic people was typically not available in this considerable population-based investigation. Besides, patients with BMI on extreme ends of the spectrum may skew the ratio and can lead to decrease sensitivity and predictive value for the NAFLD. In the future, we could design our studies to diagnose NAFLD with more appropriate methods, such as the quantitative US model and transient elastography. We could also compare TyG-BMI against liver biopsy, a definitive test to establish the diagnosis of NAFLD. Second, the authors did not receive information about the severity of hepatic steatosis, so we could not evaluate the ability of TyG-BMI to quantify hepatic steatosis. Third, in this study, the development and validation of the diagnostic value of TyG-BMI for NAFLD were conducted in Asians. The diagnostic effect of NAFLD in non-Asian populations may be limited. However, some other western-derived indices for diagnosing NAFLD, such as FLI, Framingham steatosis index (FSI), and LAP, have been validated in Asians. And they were valuable indices for identifying the presence of NAFLD [76–79]. So, we tend to believe that the TyG-BMI index could help predict NAFLD in populations other than Asians. Fourth, this was a cross-sectional study, and we could not explore the predictive value of TygBMI for the occurrence of NAFLD in the future.

## Conclusion

TyG-BMI, constructed from routine clinical and laboratory variables, is able to accurately diagnose NAFLD, thus rendering ultrasonography unnecessary for the vast majority of populations. TyG-BMI, therefore, could be used to identify candidates for hepatic ultrasound and those who need lifestyle modifications.

## Supplementary Information

**Additional file 1: Fig. S1** Distribution of TyG-BMI in the development(A) and validation groups(B)

**Additional file 2: Fig. S2** Prevalence of NAFLD according to the quartiles of TyG-BMI

**Additional file 3: Fig. S3** The ROC curve of the development group after using bootstrap resampling validation (times=500)

**Additional file 4: Fig. S4** The ROC curve of the validation group after using bootstrap resampling validation (times=500)

**Additional file 5: Fig. S5** The ROC curves of TyG-BMI in the external validation group

**Additional file 6: Fig. S6** The decision curve analysis of TyG-BMI for NAFLD in the external validation group

**Additional file 7:** **Table S1** The optimal cutoff point of 189 for the TyG-BMI in diagnosing NAFLD. **Table S2** Performance of the tests for diagnosis/exclusion of NAFLD by different subgroups in the validation group. **Table S3** Baseline characteristics of the external verification. **Table S4** Performance of the tests for diagnosing NAFLD by BMI subgroups in the development group

## Data Availability

Data can be downloaded from the ‘DATADRYAD’ database (https://datadryad.org/stash).

## Abbreviations

Ref: Reference
FPG: Fasting blood glucose
PPV: Positive predictive value
TG: Triglyceride
NPV: Negative predictive value
US: Ultrasonography
MRS: Magnetic resonance spectroscopy
MRI: Magnetic resonance imaging
HDL-c: High-density lipoprotein cholesterol
BMI: Body mass index
PLR: Positive likelihood ratio
WC: Waist circumference
NLR: Negative likelihood ratio
GGT: Gamma-glutamyl transferase
LR: Likelihood ratios
OR: Odds ratio
ROC: Receiver operating characteristic
AST: Aspartate aminotransferase
AUC: Area under the curve
TyG index: Triglyceride-glucose index
TC: Total cholesterol
SP: Specificity
DBP: Diastolic blood pressure
SN: Sensitivity
IR: Insulin resistance
AUROC: Area under the receiver-operating characteristic curve
VAI: Visceral adiposity index
LAP: Lipid accumulation product
ALT: Alanine aminotransferase
LDL-c: Low density lipoprotein cholesterol
HIS: Hepatic steatosis index;
PUFAs: Polyunsaturated fatty acids
H/R ratio: Hepatic/renal ratio
TE: Transient elastography
LSM: Liver stiffness measurement
CAP: Controlled attenuation parameter
FFAs: Free fatty acids
NASH: Non-alcoholic steatohepatitis
CI: Confidence interval
SBP: Systolic blood pressure
TyG-BMI: Triglyceride glucose-body mass index
SD: Standard deviation
NAFLD: Nonalcoholic fatty liver disease
HbA1c: Hemoglobin A1c
