# Supplementary figures and images for: The triglyceride glucose-body mass index: a non-invasive index that identifies non-alcoholic fatty liver disease in the general Japanese population
Source: J Transl Med. 2022 Sep 5;20:398. doi: 10.1186/s12967-022-03611-4 (PMC9446832; doi:10.1186/s12967-022-03611-4)

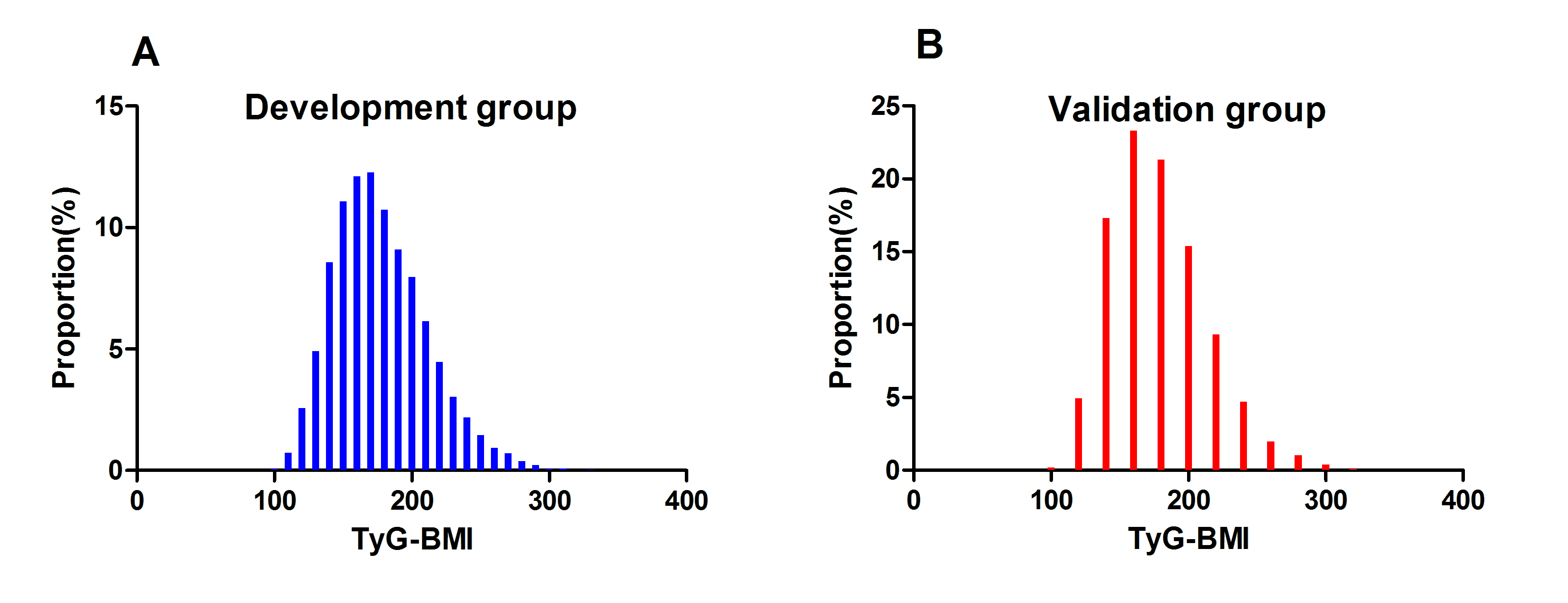

Supplement: Supplementary file 1 — Additional file 1: Fig. S1 Distribution of TyG-BMI in the development(A) and validation groups(B) [file 12967_2022_3611_MOESM1_ESM.tif]

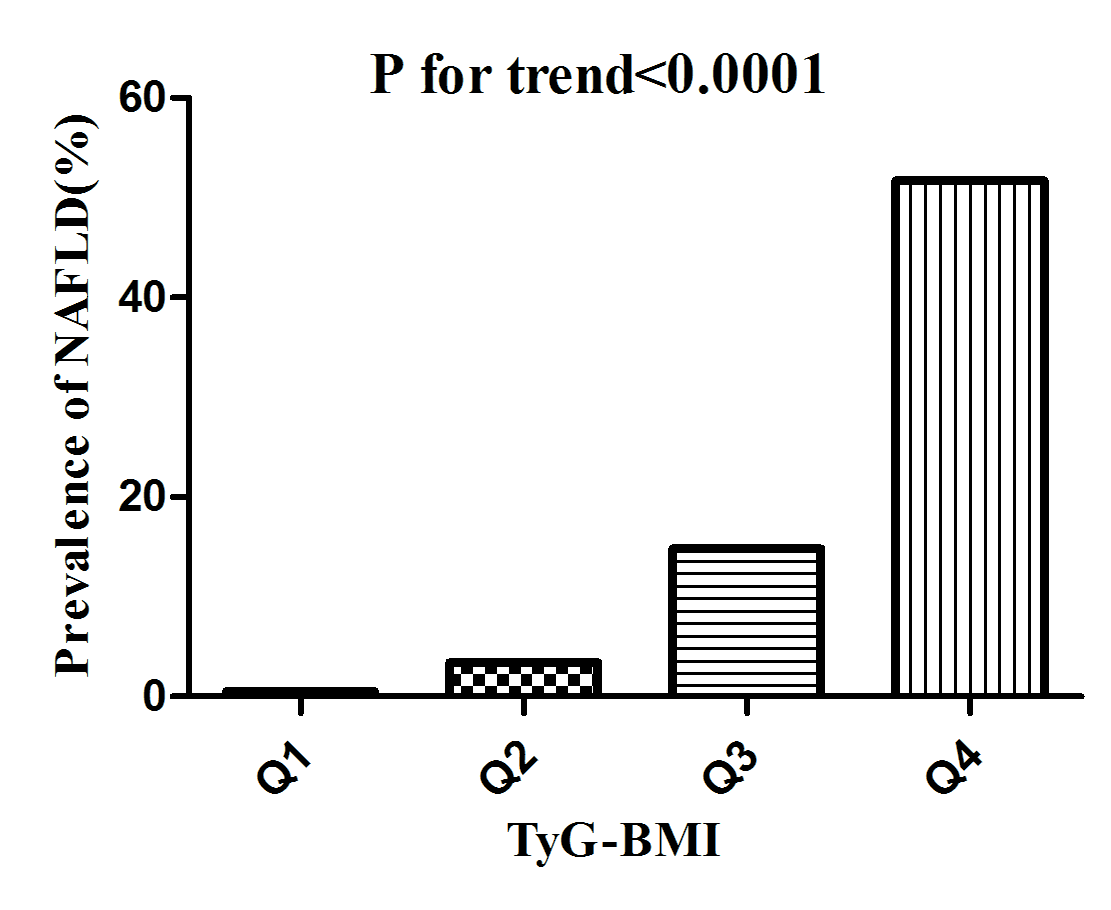

Supplement: Supplementary file 2 — Additional file 2: Fig. S2 Prevalence of NAFLD according to the quartiles of TyG-BMI [file 12967_2022_3611_MOESM2_ESM.tif]

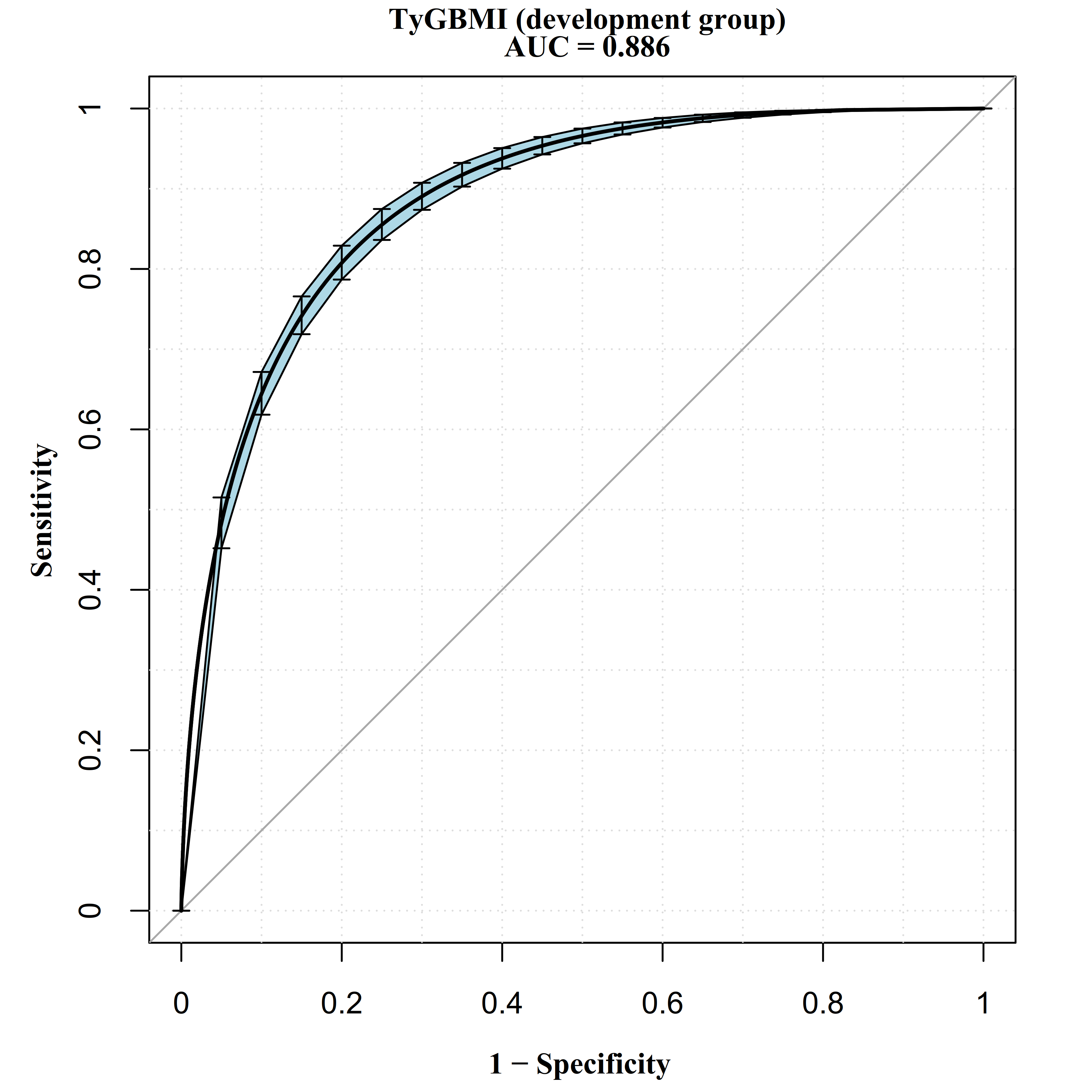

Supplement: Supplementary file 3 — Additional file 3: Fig. S3 The ROC curve of the development group after using bootstrap resampling validation (times=500) [file 12967_2022_3611_MOESM3_ESM.tif]

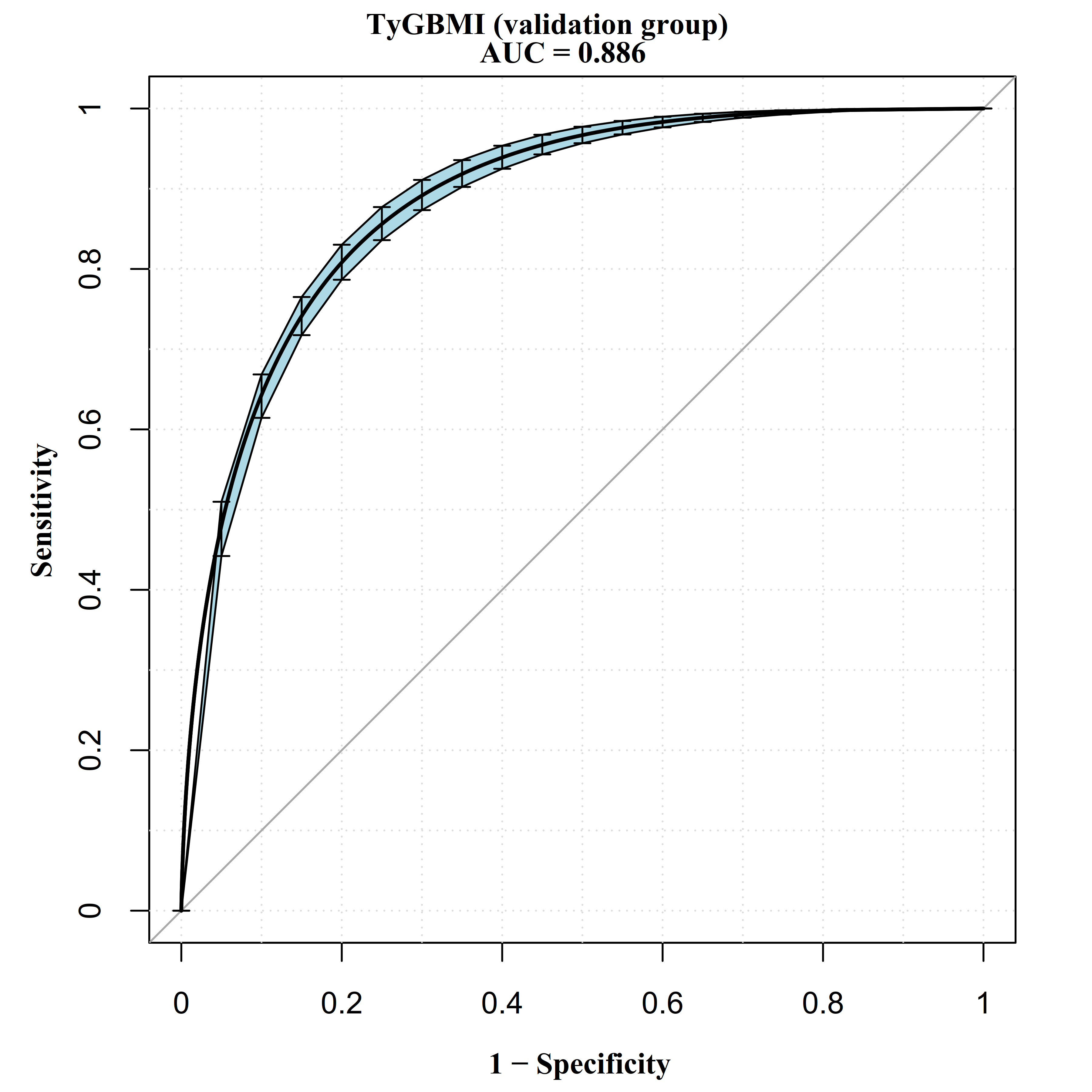

Supplement: Supplementary file 4 — Additional file 4: Fig. S4 The ROC curve of the validation group after using bootstrap resampling validation (times=500) [file 12967_2022_3611_MOESM4_ESM.tif]

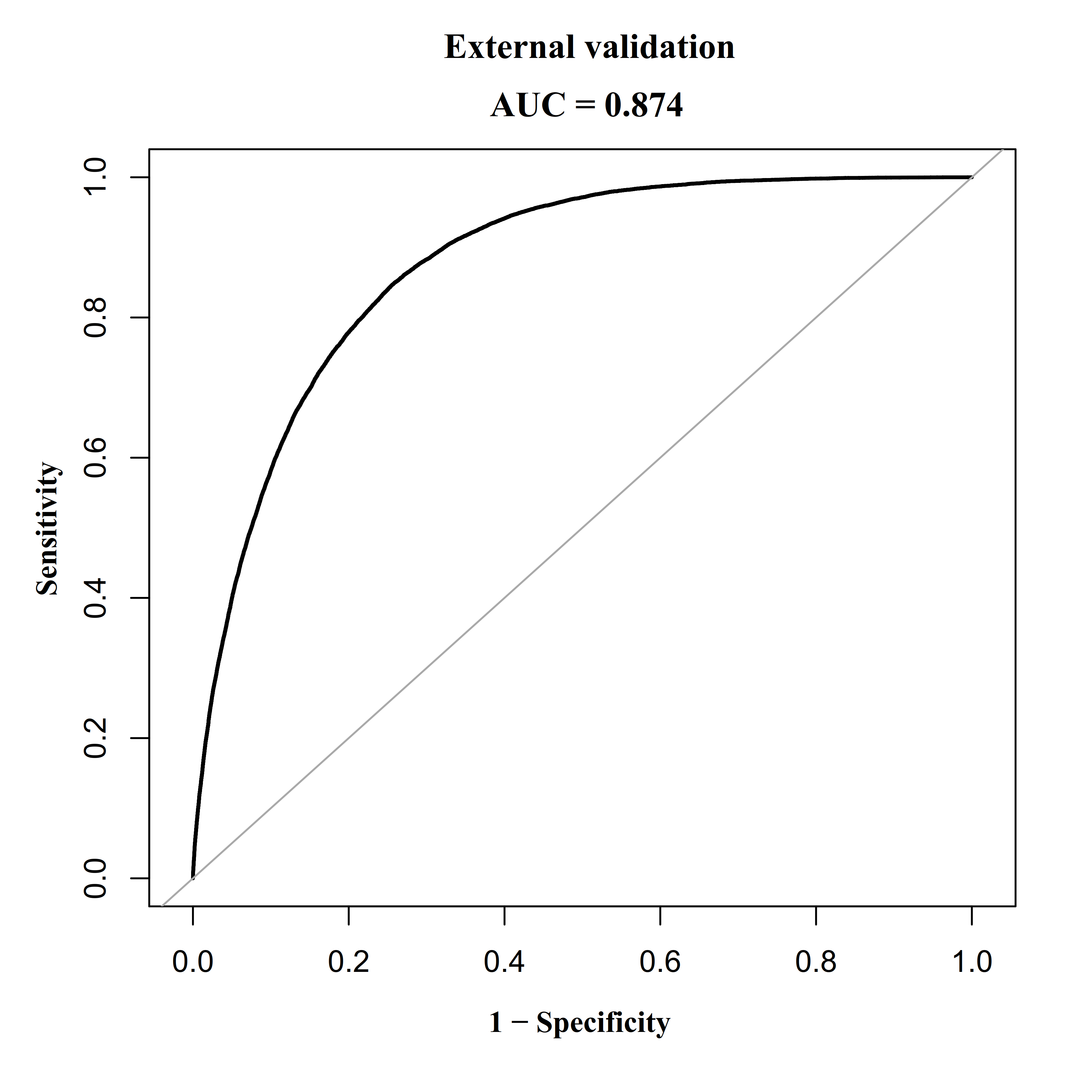

Supplement: Supplementary file 5 — Additional file 5: Fig. S5 The ROC curves of TyG-BMI in the external validation group [file 12967_2022_3611_MOESM5_ESM.tif]

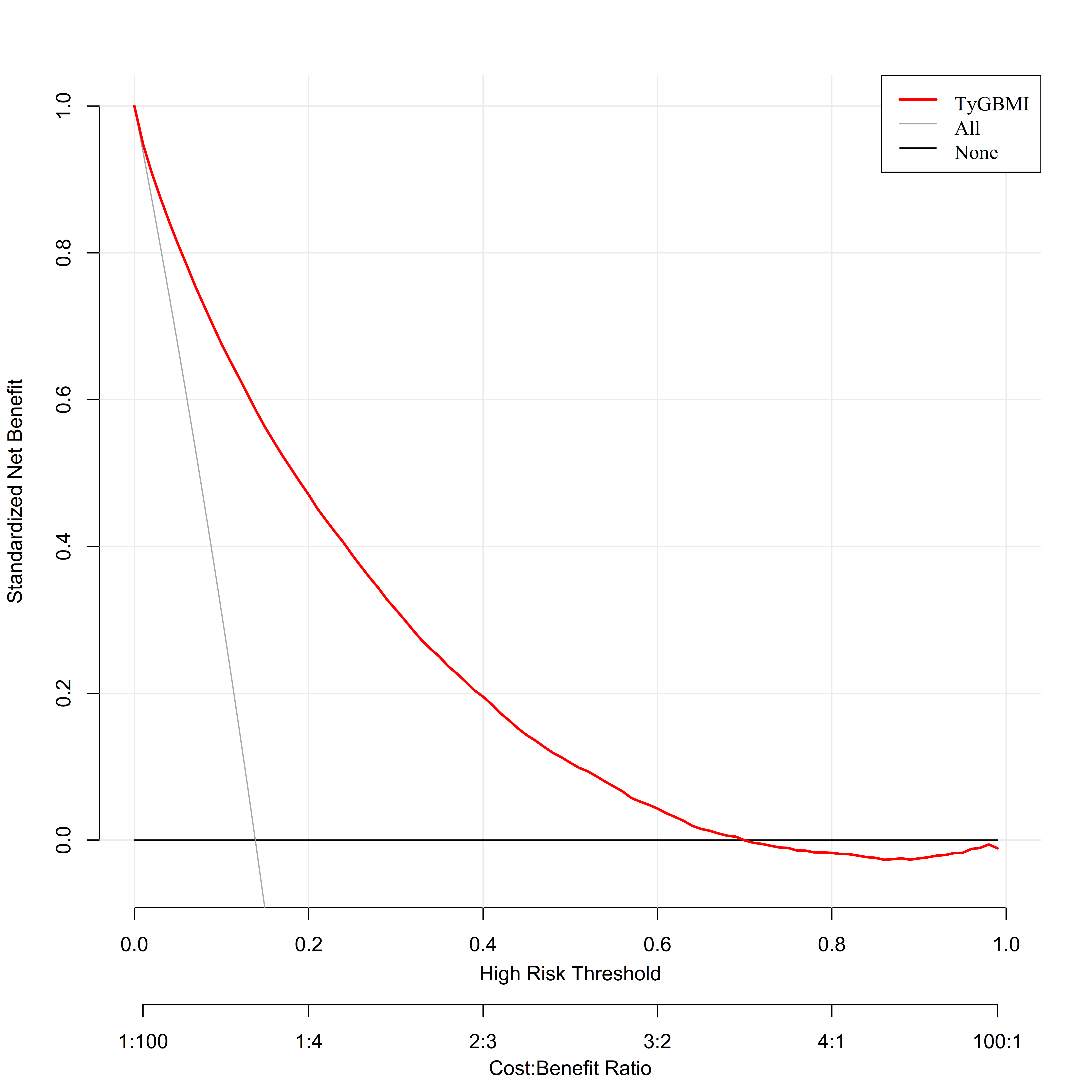

Supplement: Supplementary file 6 — Additional file 6: Fig. S6 The decision curve analysis of TyG-BMI for NAFLD in the external validation group [file 12967_2022_3611_MOESM6_ESM.tif]
