# Supplementary material for: The triglyceride glucose-body mass index: a non-invasive index that identifies non-alcoholic fatty liver disease in the general Japanese population
Source: J Transl Med. 2022 Sep 5;20:398. doi: 10.1186/s12967-022-03611-4 (PMC9446832; doi:10.1186/s12967-022-03611-4)
Supplement: Supplementary file 7 — Additional file 7: Table S1 The optimal cutoff point of 189 for the TyG-BMI in diagnosing NAFLD. Table S2 Performance of the tests for diagnosis/exclusion of NAFLD by different subgroups in the validation group. Table S3 Baseline characteristics of the external verification. Table S4 Performance of the tests for diagnosing NAFLD by BMI subgroups in the development group [file 12967_2022_3611_MOESM7_ESM.docx]

**The triglyceride glucose-body mass index: a non-invasive index that identifies NAFLD in the general Japanese population**

**Running title:** **Diagnostic value of TyGBMI for NAFLD**

**Haofei Hu^1,2,5#^, Yong Han^3,4,5#^, Changchun Cao ^6*^, Yongcheng He^7*^**

^1^Department of Nephrology, The First Affiliated Hospital of Shenzhen University, Shenzhen 518000, Guangdong Province, China

^2^Department of Nephrology, Shenzhen Second People’s Hospital, Shenzhen 518000, Guangdong Province, China

^3^Department of Emergency, Shenzhen Second People’s Hospital, Shenzhen 518000, Guangdong Province, China

^4^Department of Emergency, The First Affiliated Hospital of Shenzhen University, Shenzhen 518000, Guangdong Province, China

^5^Shenzhen University Health Science Center, Shenzhen 518071, Guangdong Province, China

^6^Department of Rehabilitation, Shenzhen Dapeng New District Nan'ao People's Hospital, Shenzhen, 518000, Guangdong Province, China.

^7^Department of Nephrology, Shenzhen Hengsheng Hospital, Shenzhen 518000, Guangdong Province, China

**^#^**Haofei Hu and Yong Han have contributed equally to this work.

*Corresponding author

**Changchun Cao**

Department of Rehabilitation

Shenzhen Dapeng New District Nan'ao People's Hospital,

No. 6, Renmin Road, Dapeng New District,

Shenzhen 518000,

Guangdong Province,

China

E-mail: caochangchun1015@163.com

**Yongcheng He,**

Department of Nephrology,

Shenzhen Hengsheng Hospital,

No. 20 Yintian Road, Baoan District,

Shenzhen 518000,

Guangdong Province,

China

Tel:+86-755-83366388

E-mail: heyongcheng640815@126.com

**Table S1. The optimal cutoff point of 189 for the TyG-BMI in diagnosing NAFLD**

| NAFLD | Development group | Validation group |
| --- | --- | --- |
| AUC | 0.8880 | 0.8845 |
| 95% CI Lower | 0.8789 | 0.8753 |
| 95% CI Upper | 0.8972 | 0.8937 |
| Best threshold | 189 | 189 |
| Specificity (%) | 77.98 | 77.38 |
| Sensitivity(%) | 84.75 | 83.51 |
| Accuracy (%) | 79.19 | 78.44 |
| PLR | 3.8496 | 3.6914 |
| NLR | 0.1956 | 0.2131 |
| PPV(%) | 45.58 | 43.67 |
| NPV(%) | 95.92 | 95.72 |

AUC, Area under the curve; CI, Confidence interval; PPV, Positive predictive value; NPV, Negative predictive value; PLR, Positive likelihood ratio; NLR, Negative likelihood ratio;

**Table S2.** **Performance of the tests for diagnosis/exclusion of NAFLD by different subgroups in the validation group**

| Validation group | AUROC (95% CI) | Cutoff | SE (%) | SP (%) | PPV (%) | NPV (%) | PLR | NLR |
| --- | --- | --- | --- | --- | --- | --- | --- | --- |
| Sex |  |  |  |  |  |  |  |  |
| Male | 0.84(0.83-0.86) | 182.2 | 91.2 | 54.6 | 42.7 | 94.3 | 2.01 | 0.16 |
|  |  | 224.0 | 44.2 | 94.3 | 74.2 | 82.0 | 7.73 | 0.59 |
| Female | 0.90(0.88-0.92) | 182.2 | 81.0 | 83.2 | 26.3 | 98.3 | 4.82 | 0.23 |
|  |  | 224.0 | 31.2 | 98.0 | 53.2 | 95.1 | 15.41 | 0.70 |
| Age |  |  |  |  |  |  |  |  |
| <30 | 0.92(0.84-0.99) | 182.2 | 80.0 | 85.9 | 24.2 | 98.7 | 5.66 | 0.23 |
|  |  | 224.0 | 50.0 | 98.3 | 62.5 | 97.2 | 29.50 | 0.51 |
| 30-40 | 0.92(0.90-0.93) | 182.2 | 89.8 | 76.4 | 38.4 | 97.9 | 3.81 | 0.13 |
|  |  | 224.0 | 49.6 | 97.2 | 74.2 | 92.2 | 17.64 | 0.52 |
| 40-50 | 0.88(0.87-0.90) | 182.2 | 89.5 | 70.0 | 40.0 | 96.7 | 2.98 | 0.15 |
|  |  | 224.0 | 40.2 | 96.1 | 70.0 | 87.8 | 10.41 | 0.62 |
| 50-60 | 0.84(0.82-0.86) | 182.2 | 88.5 | 59.2 | 38.4 | 94.7 | 2.17 | 0.19 |
|  |  | 224.0 | 38.2 | 94.8 | 68.1 | 84.2 | 7.40 | 0.65 |
| >60 | 0.83(0.77-0.88) | 182.2 | 89.5 | 60.1 | 33.1 | 96.3 | 2.24 | 0.18 |
|  |  | 224.0 | 28.1 | 95.3 | 57.1 | 85.7 | 6.04 | 0.75 |

PPV, Positive predictive value; SP, specificity; NPV, Negative predictive value; SE, Sensitivity; PLR, Positive likelihood ratio; NLR, Negative likelihood ratio; AUROC, Area under the receiver-operating characteristic curve;

**Table S3. Baseline characteristics of the external verification.**

| Characteristic | Non-NAFLD | NAFLD | P-value |
| --- | --- | --- | --- |
| N | 158243 | 25487 |  |
| Age(years) | 40.0 ± 14.0 | 46.8 ± 13.2 | <0.001 |
| GGT(U/L) | 19.0 (15.0-27.0) | 34.0 (24.0-53.0) | <0.001 |
| ALT(U/L) | 15.0 (12.0-21.0) | 25.0 (18.0-35.0) | <0.001 |
| AST(U/L) | 20.0 (18.0-24.0) | 24.0 (20.0-29.0) | <0.001 |
| ALB(g/L) | 44.5 ± 2.8 | 45.0 ± 2.8 | <0.001 |
| GLB(g/L) | 29.3 ± 3.8 | 29.5 ± 4.0 | <0.001 |
| BUN(mmol/L) | 4.4 ± 1.3 | 4.7 ± 1.2 | <0.001 |
| Scr(umol/L) | 77.7 ± 22.6 | 84.8 ± 19.3 | <0.001 |
| UA(umol/L) | 273.2 ± 84.9 | 345.0 ± 88.4 | <0.001 |
| FPG(mmol/L) | 5.1 ± 0.7 | 5.6 ± 1.3 | <0.001 |
| TC(mmol/L) | 4.5 ± 0.7 | 4.8 ± 0.8 | <0.001 |
| TG(mmol/L) | 1.0 (0.8-1.4) | 1.9 (1.3-2.7) | <0.001 |
| HDL-c(mmol/L) | 1.5 ± 0.4 | 1.3 ± 0.3 | <0.001 |
| LDL-c(mmol/L) | 2.2 ± 0.5 | 2.4 ± 0.5 | <0.001 |
| BMI (kg/m^2^) | 21.1 ± 2.1 | 23.4 ± 1.2 | <0.001 |
| TyG-BMI | 176.6 ± 23.3 | 211.4 ± 18.7 | <0.001 |
| Sex, n(%) |  |  | <0.001 |
| Female | 86831 (54.9%) | 5701 (22.4%) |  |
| Male | 71412 (45.1%) | 19786 (77.6%) |  |

Values are n(%) or mean±SD or medians (quartiles)

BMI, Body mass index; GGT, *γ*-glutamyl transpeptidase; AST, Aspartate aminotransferase; TG, Triglyceride; ALB, albumin; ALT, Alanine aminotransferase; GLB, globulin; LDL-C, Low-density lipid cholesterol; BUN, Serum urea nitrogen; HDL-c, High-density lipoprotein cholesterol; Scr, Serum creatinine; TC, Total cholesterol; FPG, Fasting plasma glucose; UA, uric acid; TyG-BMI: Triglyceride glucose-body mass index;

**Table S4. Performance of the tests for diagnosing NAFLD by BMI subgroups in the development group.**

| TyG-BMI | AUC (95%CI) | Best threshold | SP (%) | SE (%) | PPV (%) | NPV (%) | PLR | NLR |
| --- | --- | --- | --- | --- | --- | --- | --- | --- |
| BMI<24 | 0.84 (0.83-0.86) | 177.24 | 76.04 | 77.78 | 21.49 | 97.59 | 3.25 | 0.29 |
| BMI≥24 | 0.74 (0.72-0.76) | 220.16 | 70.96 | 63.72 | 69.18 | 65.66 | 2.19 | 0.51 |

**Figure S1. Distribution of TyG-BMI in the development(A) and validation groups(B).**

Figure S1 showed that TyG-BMI were distributed normally both in the development and validation groups.

**Figure S2. Prevalence of NAFLD according to the quartiles of TyG-BMI.**

Figure S2 indicated that participants in the high TyG-BMI group had a higher NAFLD prevalence than the lowest TyG-BMI group (p<0.0001 for trend).

Classification of TyG-BMI quartiles: Q1 (~152.2), Q2 (152.3 ~ 173.2), Q3 (173.3~ 198.3), Q4 (198.4~). Q1, first quartile; Q2, second quartile; Q3, third quartile; Q4, fourth quartile

**Figure S3. The ROC curve of the development group after using bootstrap resampling validation (times=500)**

Figure S3 showed that using 500 bootstrap resamplings, TyG-BMI had an average AUC of 0.886 (95% CI 0.876, 0.896). The AUROC remained high and almost unchanged in the development set.

**Figure S4. The ROC curve of the validation group after using bootstrap resampling validation (times=500)**

Figure S4 showed that using 500 bootstrap resamplings, TyG-BMI had an average AUC of 0.886 (95% CI 0.877, 0.897). The AUROC remained high and almost unchanged in the validation set.

**Figure S5. The ROC curves of TyG-BMI in the external validation group.**

Figure S5 showed that the AUC of the external validation was 0.874.

**Figure S6. The decision curve analysis of TyG-BMI for NAFLD in the external validation group.**

Figure S6 showed that TyG-BMI had an excellent clinical application value for diagnosing or excluding NAFLD in the external validation group.

The black line represents the net benefit when none of the participants are considered to develop NAFLD. In contrast, the light gray line represents the net benefit when all participants are considered to develop NAFLD. The area between the "no treatment line" (black line) and "all treatment line" (light gray line) in the model curve indicates the clinical utility of the model. The farther the model curve is from the black and light gray lines, the better the clinical use of the model.
